# Selectivity and robustness of sparse coding networks

**DOI:** 10.1167/jov.20.12.10

**Published:** 2020-11-25

**Authors:** Dylan M. Paiton, Charles G. Frye, Sheng Y. Lundquist, Joel D. Bowen, Ryan Zarcone, Bruno A. Olshausen

**Affiliations:** 1 Vision Science Graduate Group, University of California Berkeley, Berkeley, CA, USA; 2 Redwood Center for Theoretical Neuroscience, University of California Berkeley, Berkeley, CA, USA; 3 Helen Wills Neuroscience Institute, University of California Berkeley, Berkeley, CA, USA; 4 Department of Computer Science, Portland State University, Portland, OR, USA; 5 Biophysics, University of California Berkeley, Berkeley, CA, USA

**Keywords:** sparse coding, lateral inhibition, orientation selectivity, visual cortex, robustness

## Abstract

We investigate how the population nonlinearities resulting from lateral inhibition and thresholding in sparse coding networks influence neural response selectivity and robustness. We show that when compared to pointwise nonlinear models, such population nonlinearities improve the selectivity to a preferred stimulus and protect against adversarial perturbations of the input. These findings are predicted from the geometry of the single-neuron iso-response surface, which provides new insight into the relationship between selectivity and adversarial robustness. Inhibitory lateral connections curve the iso-response surface outward in the direction of selectivity. Since adversarial perturbations are orthogonal to the iso-response surface, adversarial attacks tend to be aligned with directions of selectivity. Consequently, the network is less easily fooled by perceptually irrelevant perturbations to the input. Together, these findings point to benefits of integrating computational principles found in biological vision systems into artificial neural networks.

## Introduction

Inhibitory lateral connections abound in biological neural networks. In the visual system, they are found in the retina, LGN, and nearly all layers of visual cortex. In the retina, horizontal cells provide inhibitory feedback onto photoreceptors, performing a form of spatial differentiation that is thought to reduce redundancy in the signals sent down the optic nerve (Srinivasan, Laughlin, & Dubs, 1982; van Hateren, 1992; Atick & Redlich, 1990, 1992). In the lateral geniculate nucleus, inhibitory interneurons are thought to mediate spatial and temporal sharpening of image contrast (Hirsch, Wang, Sommer, & Martinez, 2015). In primary visual cortex, inhibitory lateral connections have been implicated as a mechanism responsible for nonlinear response properties such as divisive normalization, cross-orientation inhibition, and contrast-invariant orientation tuning (Carandini, Heeger, & Movshon, 1997; Zetzsche, Krieger, & Wegmann, 1999; Douglas & Martin, 2007; Priebe & Ferster, 2012; Zhu & Rozell, 2013).

In contrast to this pervasive feature of neurobiological networks, the deep neural network architectures that are now widely used for image analysis (LeCun, Bengio, & Hinton, 2015; Rawat & Wang, 2017; Goodfellow, Bengio, & Courville, 2016) and proposed as models of the visual system (Yamins, Hong, Cadieu, Solomon, Seibert, & DiCarlo, 2014; Doi & Lewicki, 2014; Yamins & DiCarlo, 2016; Lindsey, Ocko, Ganguli, & Deny, 2019; Richards et al., 2019) utilize only a cascade of linear filtering followed by pointwise nonlinearities (e.g., rectification) at each stage of processing. Here we ask what could be gained by incorporating the *population nonlinearities* that arise from recurrent inhibition within the lamina of visual cortex (Xu, Olivas, Ikrar, Peng, Holmes, Nie, & Shi, 2016). An important computational property of these recurrent inhibitory networks, in comparison to a layer of neurons within a feedforward network, is that neurons can increase their selectivity by recirculating information within the same layer rather than relying upon additional downstream layers of processing, thus making more efficient use of neural resources. We focus here specifically on the form of lateral inhibition proposed by the sparse coding model, which hypothesizes that cortical networks achieve sparse representations via neurons inhibiting each other proportional to the overlap in their receptive fields (Olshausen & Field, 1996; Rozell, Johnson, Baraniuk, & Olshausen, 2008), an idea that is both theoretically grounded and empirically supported (Zetzsche & Krieger, 1999; Olshausen & Field, 2004; Haider, Krause, Duque, Yu, Touryan, Mazer, & McCormick, 2010; Chettih & Harvey, 2019; Beyeler, Rounds, Carlson, Dutt, & Krichmar, 2019). We show how these interactions give rise to both a higher degree of *selectivity* and increased *robustness* in comparison to the purely feedforward network layers lacking such interactions.

Our analysis characterizes the response properties of model neurons in terms of their iso-response surface, that is, the surface in stimulus space defined by the set of stimuli that produce equal responses from a neuron. These surfaces can be curved for single-layer networks with population nonlinearities, such as sparse coding, and are always flat for single-layer networks with pointwise nonlinear activation functions, such as those composed of linear nonlinear poisson (LNP) neurons (Zetzsche & Röhrbein, 2001; Golden, Vilankar, Wu, & Field, 2016). Iso-response stimulus analysis has been used to better understand neural computation for visual (Rust, Schwartz, Movshon, & Simoncelli, 2005; Bölinger & Gollisch, 2012; Horwitz & Hass, 2012) and auditory (Gollisch & Herz, 2005) brain processing regions (for a review, see Gollisch & Herz, 2012). Previous work has suggested that curved iso-response contours are indicative of a multiplicative AND-like operation on the inputs, resulting in improved efficiency and selectivity (Zetzsche & Barth, 1990; Zetzsche & Krieger, 2001). Building on these ideas, Vilankar & Field (2017) defined *hyperselectivity* as the drop-off in response around a neuron's preferred stimulus and explored its relation to the iso-response curvature of sparse coding neurons. Here, we extend the analysis to provide a more complete description of the curvature for a large sample of neurons in network models trained on a data set of natural images. We then use experimental designs adapted from neurophysiological studies using full-field grating stimuli (Ringach, Shapley, & Hawken, 2002) as well as natural stimuli to show that the hyperselectivity of neurons in a sparse coding network results in sharper tuning than in linear or pointwise nonlinear neurons.

The drop-off in a neuron's response around its preferred stimulus can conversely be thought of as robustness against perturbations that are not aligned with the preferred stimulus direction. The lack of robustness in deep neural networks has been a topic of great interest to the machine learning community. It has been shown that these networks are easily fooled by small perturbations designed to maximally change the network's output while minimally changing the input (Szegedy et al., 2013) or even by real-world photos that fall outside the traditional training/test ensemble (Hendrycks, Zhao, Basart, Steinhardt, & Song, 2019; Recht, Roelofs, Schmidt, & Shankar, 2019). While we recognize the importance of the training loss, here we provide evidence to support the hypothesis that the observed lack of robustness is in part due to the manner in which these networks were constructed to begin with, that is, as a passive, feedforward cascade of filtering and pointwise nonlinearities. By contrast, sparse coding uses a probabilistic, generative model that attempts to explain what it “sees” in terms of a model of the world (Olshausen, 2013b). Importantly, the representation of an image is inferred through a dynamic process that compares the model's prediction against the data, inducing an “explaining away” competition among neurons (Pearl, 1988). As we shall see, this causes the neurons to have iso-response surfaces that are curved outward, away from the origin and in the direction of selectivity, hence providing hyperselectivity. Consequently, the response to a stimulus that is not aligned with a neuron's weight vector will be attenuated. Here we show how this hyperselectivity makes neurons more resistant to adversarial perturbations.

We first review locally competitive algorithms (LCAs; Rozell et al., 2008), a family of recurrent neural networks with lateral connections for implementing sparse coding, which forms the basis of our investigation. Next we characterize the iso-response surface and measure selectivity to orientated gratings as well as natural stimuli for networks with and without lateral connections. We demonstrate the relation between selectivity and robustness with an analytic argument and show that the curved iso-response surfaces resulting from lateral inhibition encourage adversarial perturbation vectors to be more aligned with the data dimensions and thus more semantically relevant. Finally, we demonstrate by experiment improved robustness to adversarial attacks with networks that include lateral inhibitory connections. We contribute a novel perspective for relating iso-response surfaces, selectivity, and adversarial robustness. Some of this work has been previously described in (Paiton, 2019), although here we provide more complete analysis, interpretation, and additional experiments.

## Neuron response geometry

### Sparse coding

Sparse coding is a generative model for representing natural stimuli (Olshausen and Field, 1997). The model aims to encode an incoming signal efficiently under the assumption that it is composed of structured components and unstructured additive noise. It assumes a linear generative model:

(1) $$\begin{array}{c} s=\Phi a+ɛ,\; \end{array}$$

where $s\in R^{P}$ is a vector of P image pixels, $a\in R^{N}$ is a vector of N neuron activation coefficients, Φ is a P×N dictionary matrix (the structured components), and $ɛ\in R^{P}$ is Gaussian noise. Importantly, to encode a given input, s, the model must infer appropriate coefficients, a, as opposed to directly computing them with a feedforward process. The encoding should be a faithful and efficient representation of the data, which is achieved by minimizing an energy function:

(2) $$\begin{array}{c} \underset{a}{\mathrm{argmin}}\left(E=\frac{1}{2}{∥s-\hat{s}∥}_{2}^{2}+\lambda \sum_{i=1}^{N}C\left(a_{i}\right)\right),\; \end{array}$$

where $\hat{s}=\sum_{i=1}^{N}\Phi_{i}a_{i}$ is the image reconstruction, λ trades off the reconstruction accuracy against network sparsity, and C(·) is the sparsity constraint cost function. In some experiments, we will vary the degree of overcompleteness of Φ, which is represented by the ratio $\frac{N}{P}$.

Rozell et al. (2008) proposed a family of dynamical neural networks called LCAs or LCA for a single network type) to minimize Equation (2). LCAs describe each activation coefficient, $a_{k}$, as the thresholded output of an internal state variable, $u_{k}$, which is analogous to a biological neuron's membrane potential and evolves according to the following differential equation:

(3) $$\begin{array}{c} \dot{u_{k}}\left(t\right)=\frac{1}{\tau }\left[b_{k}-\sum_{n\neq k}^{N}G_{k,n}a_{n}\left(t\right)-u_{k}\left(t\right)\right],\; \end{array}$$

where τ represents the time constant of the dynamics, $b_{k}=\Phi_{k}^{⊤}\;s$ is the feedforward drive, and $G_{i,j}=\Phi_{i}^{⊤}\;\Phi_{j}$ is an entry in the lateral connectivity matrix. The relation between $a_{k}\left(t\right)$ and $u_{k}\left(t\right)$ is given by

(4) $$\begin{array}{ccc} a_{k}\left(t\right) & \;= & T_{\lambda }\left(u_{k}\left(t\right)\right)\\ T_{\lambda }\left(u_{k}\left(t\right)\right) & \;= & \left\{\begin{array}{cc} 0,\;\; & u_{k}\left(t\right)\;\leq \;\lambda \\ u_{k}\left(t\right)-\lambda ,\;\; & u_{k}\left(t\right)\;>\;\lambda . \end{array}\right.\; \end{array}$$

Note that our $T_{\lambda }\left(\cdot \right)$ is a nonnegative variant of what was specified by Rozell et al. (2008). Other thresholding functions can be derived for different choices of the cost function $C\left(\cdot \right)$ (Rozell et al., 2008; Charles, Garrigues, & Rozell, 2011). In this work, we employ a single instance of the family of networks that implements an $l_{1}$ sparseness penalty: $C\left(a_{i}\right)=\left|a_{i}\right|$, although we expect other sparsity-inducing choices would yield similar results. For all of the experiments in this study, we pre-trained the LCA weights by minimizing the energy function in Equation (2) with respect to Φ via the learning rule

(5) $$\begin{array}{c} \Delta \Phi_{k}=\eta \left(s-\hat{s}\right)a_{k},\; \end{array}$$

where η is the learning rate, the actual weight update is the average over a batch of 100 inputs, and $a_{k}$ indicates the activation after T update steps, $a_{k}\left(t=T\right)$.

**Figure 1.**
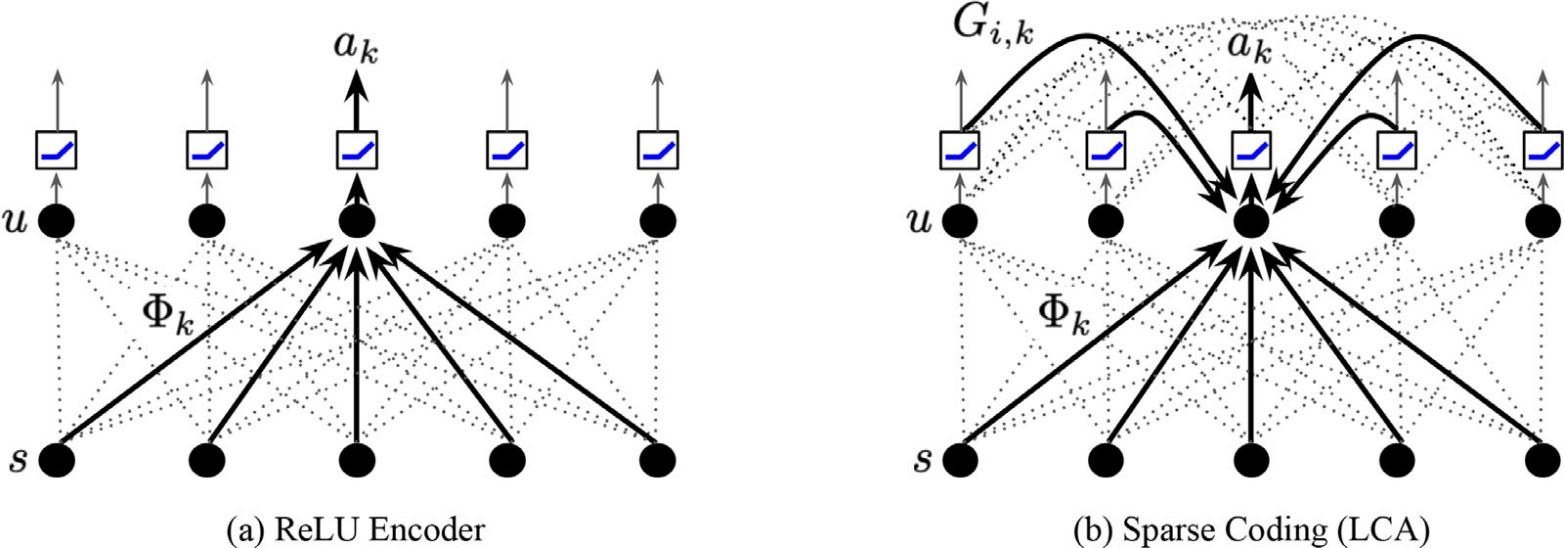
Network architectures. The input, s, is a vector of pixels represented by the lower row of black dots. The neurons are the upper row of black dots and have internal states, u. The dark arrows are connections for neuron k. All other connections are denoted by dotted lines. (a) A standard architecture for a feedforward rectified (ReLU) encoder. (b) The architecture for the LCA network, which includes feedforward driving connections, Φ, as well as a lateral connectivity matrix, G.

The dynamics of Equations (3) and (4) correspond to a recurrent neural network, where each unit is driven by a feedforward component given by the similarity between its dictionary element and the signal, and inhibited by lateral connections that have strength proportional to the overlap in units’ feedforward weights, as shown in Figure 1b. In contrast to a standard neural network layer composed of linear summation followed by pointwise nonlinearities, shown in Figure 1a, the LCA network expresses a *population nonlinearity* as the nonlinear mapping between s and a is a function of the whole layer of neurons. In the next section, we compare the iso-response surfaces that result from these two different network architectures.

### Characterizing neurons via iso-response surfaces

The activation of a single model neuron is a scalar-valued function, f, of a vector-valued input, $s\in R^{P}$. The set of all vectors that are mapped by f to the same response value c is known as the level set at c of f. For a given input s such that f(s)=c, we call the connected component of the level set at c that contains s the *iso-response surface* of f at s. It is the set of all inputs obtainable by a smooth transformation of s that are mapped to the same output value c. This surface is generically P-1 dimensional. In order to better understand and to visualize this high-dimensional object, we consider its lower-dimensional projections. We call a parameterized curve, γ(t), along this surface and including the point s an *iso-response curve* at s. An *iso-response contour* at s is an iso-response curve at s that is restricted to a two-dimensional subspace of $R^{P}$. Alternatively, it is the iso-response surface of the activation function restricted to this subspace.

**Figure 2.**
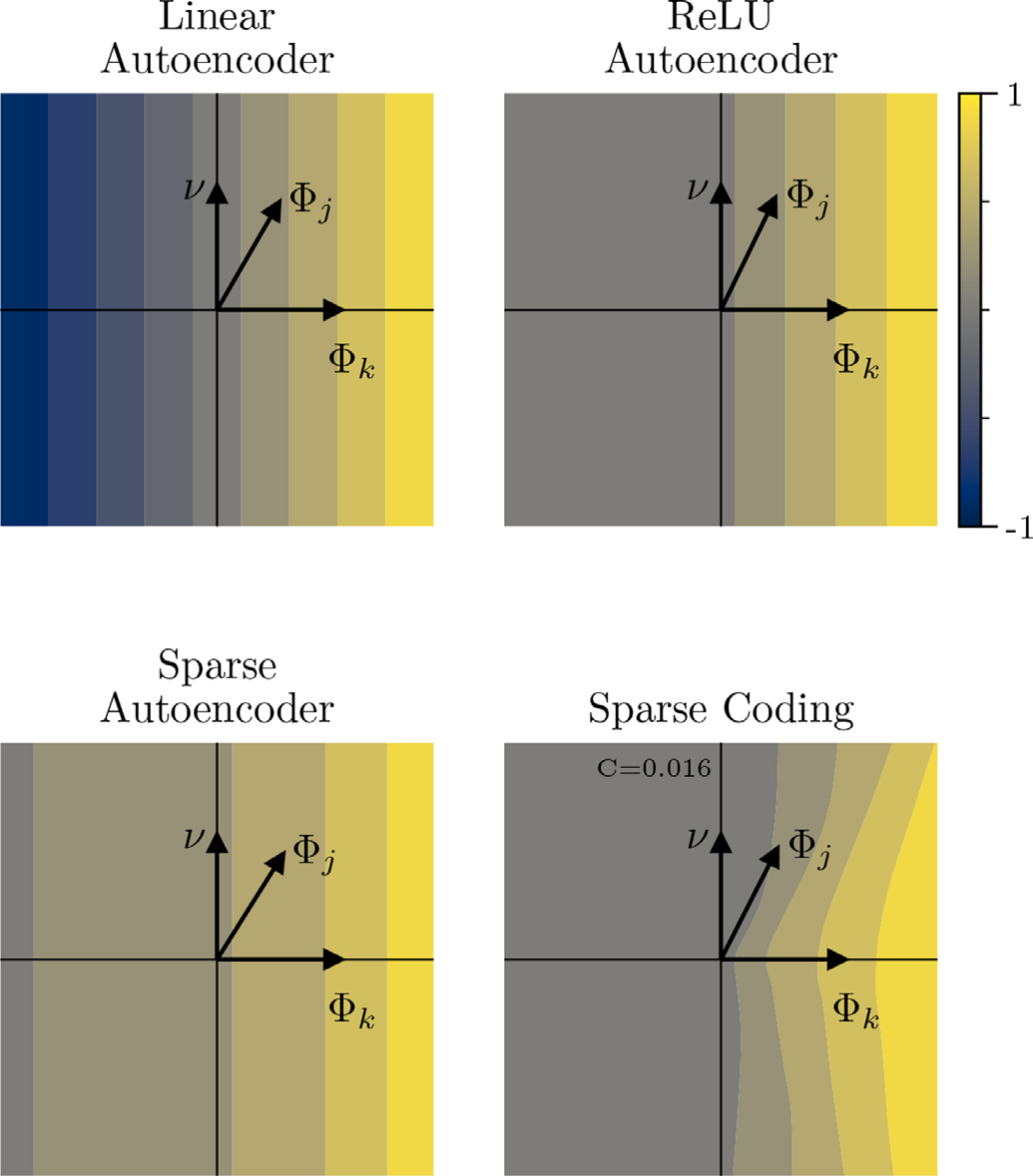
Empirically measured iso-response contours. A fine sampling of points in a two-dimensional plane is injected into a 256-dimensional image space and used as inputs to a target model neuron. For each subplot, neuron k's outputs for all images were normalized and then divided into 10 bins, which are indicated by the color. Weight vectors for neurons $\Phi_{k}$ and $\Phi_{j}$ are shown, although the Φ matrix differs from model to model. ν indicates an orthogonal vector found using the orthogonalization process described in the text. All pointwise nonlinear models will produce straight contours that are orthogonal to the $\Phi_{k}$ weight vector, while the population nonlinear model can produce exo-origin bent (i.e., bent away from the origin) contours. C indicates curvature, which is measured using the method discussed in Appendix A.3.

To visualize a target neuron's iso-response contours, we measure the neuron's response to a data set of images that all lie on a two-dimensional subspace of $R^{P}$, shown in Figure 2. We use the target neuron's feedforward weights $\Phi_{k}$ as one of the two vectors that define the subspace. To determine the second axis of the subspace, we start by choosing a random comparison neuron with a weight vector ($\Phi_{j},j\neq k$). In the likely event that the comparison vector is not orthogonal to the target vector, we use one step of the Gram-Schmidt process to find an orthogonal vector that is coplanar with the comparison and target neurons. As opposed to randomly selecting the orthogonal direction, this method will increase the likelihood of competition between neurons for LCA networks and thus increase the curvature (Golden et al., 2016; Vilankar & Field, 2017). Each point within a reasonable radius (given the norms of the weight vectors and training stimuli) of the origin in the two-dimensional plane can be injected into $R^{P}$ to produce images that will have a high degree of correspondence to features that are relevant to the target neuron (although this is most true for the upper-right quadrant, we assume it is approximately also true for the rest of the quadrants). Finally, we bin the points according to the target neuron's normalized output amplitude so that the bin boundaries reveal the neuron's iso-response contours.

Now let us consider a linear neuron model. The iso-response contours of linear neurons are straight: Any input perturbation that is orthogonal to the weight vector will result in equal activation. Writing s for the input and e for the perturbation, we have

(6) $$\begin{array}{c} \Phi_{k}^{⊤}\left(s+e\right)=\Phi_{k}^{⊤}s+\Phi_{k}^{⊤}e.\; \end{array}$$

This will be constant for perturbations, e, such that $\Phi_{k}^{⊤}e=0$. These perturbations are orthogonal to $\Phi_{k}$ or, more generically, in the N-1 dimensional nullspace of the linear map $\Phi_{k}^{⊤}$. Therefore, the activation of the neuron is constant in a linear subspace of dimension P-1, or a hyperplane, and all of its iso-response contours are straight and orthogonal to the weight vector (see Figure 2, top left).

Pointwise nonlinearities are the more traditional form of nonlinearities and are seen in many deep neural network architectures and computational neuroscience models. They can be defined as nonlinearities that are a function of only a single neuron in a layer and include rectification, sigmoid, and hyperbolic tangent (among other functional variants). Pointwise nonlinearities also produce straight iso-response contours because the nonlinearity is performed after a linear projection. Writing g for the nonlinearity of the neuron k, we have

(7) $$\begin{array}{c} g\left(\Phi_{k}^{⊤}\left(s+e\right)\right)=g\left(\Phi_{k}^{⊤}s+\Phi_{k}^{⊤}e\right),\; \end{array}$$

which is once again constant for orthogonal perturbations, $\Phi_{k}^{⊤}e=0$ (see Figure 2, top right and bottom left).

Population nonlinearities represent an alternative class of nonlinearities, where the output is also a function of multiple neurons in a set. These include divisive normalization (e.g., Geisler & Albrecht, 1992; Carandini & Heeger, 2012; Sanchez-Giraldo, Laskar, & Schwartz, 2019) and the network nonlinearity, present in sparse coding. By contrast, for a population nonlinearity, the gradient of the activation with respect to a small perturbation in the input is a function of all other neurons in the layer. Consider: for a perturbation that is orthogonal to a target neuron's weight vector, it is generically the case that some other neuron will have a nonorthogonal weight vector, which can result in a net change in all neuron outputs. Writing g for the population nonlinearity and $p_{k}$ for the kth canonical basis vector (i.e., a one-hot vector that selects neuron k), the activation of a neuron k can be written

(8) $$\begin{array}{c} p_{k}^{⊤}g\left(\Phi^{⊤}\left(s+e\right)\right)=p_{k}^{⊤}g\left(\Phi^{⊤}s+\Phi^{⊤}e\right),\; \end{array}$$

where the term inside g is again constant along linear spaces in a nullspace, in this case that of the weight matrix. When the output layer has more neurons than the input layer, as in overcomplete sparse coding, this nullspace only contains the zero vector, and therefore, g will not be constant along any linear subspace. In this case, the iso-response contours for population nonlinear neurons will generically be curved (Figure 2, bottom right). The curvature can be toward the origin (endo-origin) or away from it (exo-origin). Our experiments herein as well as work from others support the hypothesis that exo-origin curvature is indicative of general selectivity—there will be a drop-off of the neuron's response when the input is perturbed away from its preferred stimulus (Zetzsche & Röhrbein, 2001; Vilankar & Field, 2017). We focus on a single method for implementing population nonlinearities, and in the Discussion, we point to several alternative approaches that warrant additional comparisons.

### Population iso-response surface analysis

**Figure 3.**
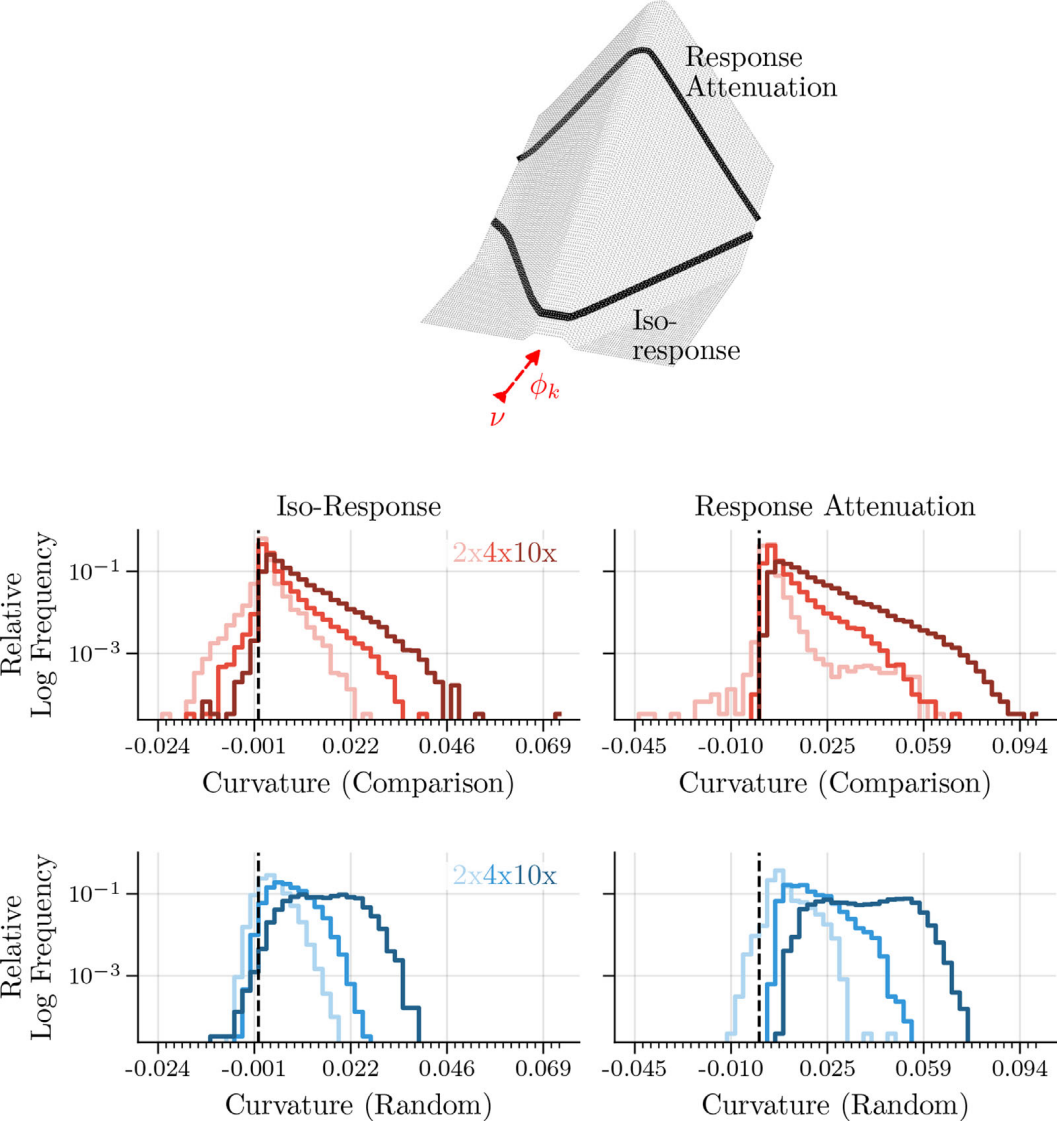
LCA neurons have high-dimensional exo-origin curvature. The top plot is a three-dimensional response surface plot, where the color axis used in Figure 2 is now indicated by the z-axis. The y- and x-axes are indicated by projections of the ν and $\Phi_{k}$ vectors, respectively. Two different types of curvature indicated, which are dependent on each other but not equal. The histograms show second-order coefficients for polynomial fits to points measuring (left column) iso-response curves and (right column) response attenuation curves. The black vertical dashed lines indicate 0 curvature and color darkness indicates the network overcompleteness. See text for details about comparison (red lines) versus random (blue lines) orthogonal vectors. We plot the logarithm of the frequency to emphasize the behavior of the tails, although we provide a linear version in Figure A.2.

By observing many individual response contours, we can gain a better intuition about the higher-dimensional response surface. We do this by calculating the response contours for different two-dimensional cross sections of the P-dimensional image space and then summarizing the estimated curvature in all of the observed planes. As described earlier, we define one axis of all planes as the target neuron's weight vector. Next, we propose two different methods for finding the orthogonal axis. The first method, which we call the “comparison” plane method in Figure 3, is to iteratively apply the process we described above for a large sampling of other neurons in the layer. Specifically, we select 300 comparison neuron vectors randomly from the set of alternative neuron weights for each target vector. This analysis method is general in that one could have used most-exciting images or any other variety of stimuli to define the planes, although for understanding selectivity and robustness of single-layer networks, we found that the feedforward weights are the most interpretable choice. For the second method, which we call the “random” plane method in Figure 3, we compute a set of planes defined by random orthogonal vectors that are also orthogonal to $\Phi_{k}$. This method will result in less curvature but provides a more complete description of the high-dimensional response geometry. Since our “random” plane selection method still uses the neuron's weight for one axis, the likelihood of competition (and therefore curvature) is higher than if both axes were chosen randomly. This is because the angles between LCA weight vectors are much more diffusely distributed around orthogonal than they would be for random vectors (Vilankar & Field, 2017; Paiton, 2019). For each plane, we compute the model outputs for 900 inputs evenly spaced in a two-dimensional grid pattern centered on the origin. The first method is the same as what was used by Vilankar & Field (2017), although they measure curvature for single-neuron pairs. To better understand the response geometry of the entire network, we analyze 100 randomly selected neurons and 600 orthogonal planes per neuron (300 per method), resulting in 54 million image presentations per overcompleteness level.

In addition to the curvature of iso-response lines, it is also relevant to measure the curvature of response attenuation lines, which are orthogonal to the target neuron's weight vector (Figure 3, top). This type of curvature indicates how much a neuron's response decreases as the stimulus becomes less like its weight vector and is a direct measure of selectivity against orthogonal perturbations. For pointwise nonlinear neurons, these two lines have zero curvature, and for population nonlinear neurons, it is possible for them to have different curvatures.

We measured the curvature of the two contour types (iso-response and response attenuation) in each plane for all neurons tested using the method described in Appendix A.3. Figure 3 demonstrates that LCA neurons have exo-origin iso-response curvature and response attenuation curvature in nearly all data-relevant planes. The high-dimensional curvature for LCA neurons can be thought of as an irregular hyper-cone, which indicates selectivity against perturbations away from its feedforward receptive field. This is an important quality that we desire from our model neurons. In visual neuroscience, we often use the neuron's linear receptive field (in our model that is analogous to its weight vector) to represent the stimulus that the neuron is selective for. With a pointwise nonlinear neuron model, it is possible to deviate far away from its weight vector in any orthogonal direction without changing the neuron's response. LCA neurons, on the other hand, have a higher degree of selectivity to perturbations away from their receptive field. Therefore, neurons with exo-origin response curvature produce outputs with a higher degree of correspondence to what we believe they are looking for in the world. The link between exo-origin iso-response curvature and selectivity has been shown experimentally (Horwitz & Hass, 2012; Bölinger & Gollisch, 2012) as well as argued theoretically (Zetzsche & Röhrbein, 2001; Vilankar & Field, 2017). We expand on previous work in Figure 3 by showing that the amount of response curvature is increased as one increases model overcompleteness for a large sample of population nonlinear neurons. In the following section, we draw additional connections by showing improved orientation and natural stimuli selectivity for LCA neurons when compared to linear and pointwise nonlinear neuron models, which we argue is predicted by the response curvature.

## Selectivity

### Orientation selectivity

**Figure 4.**
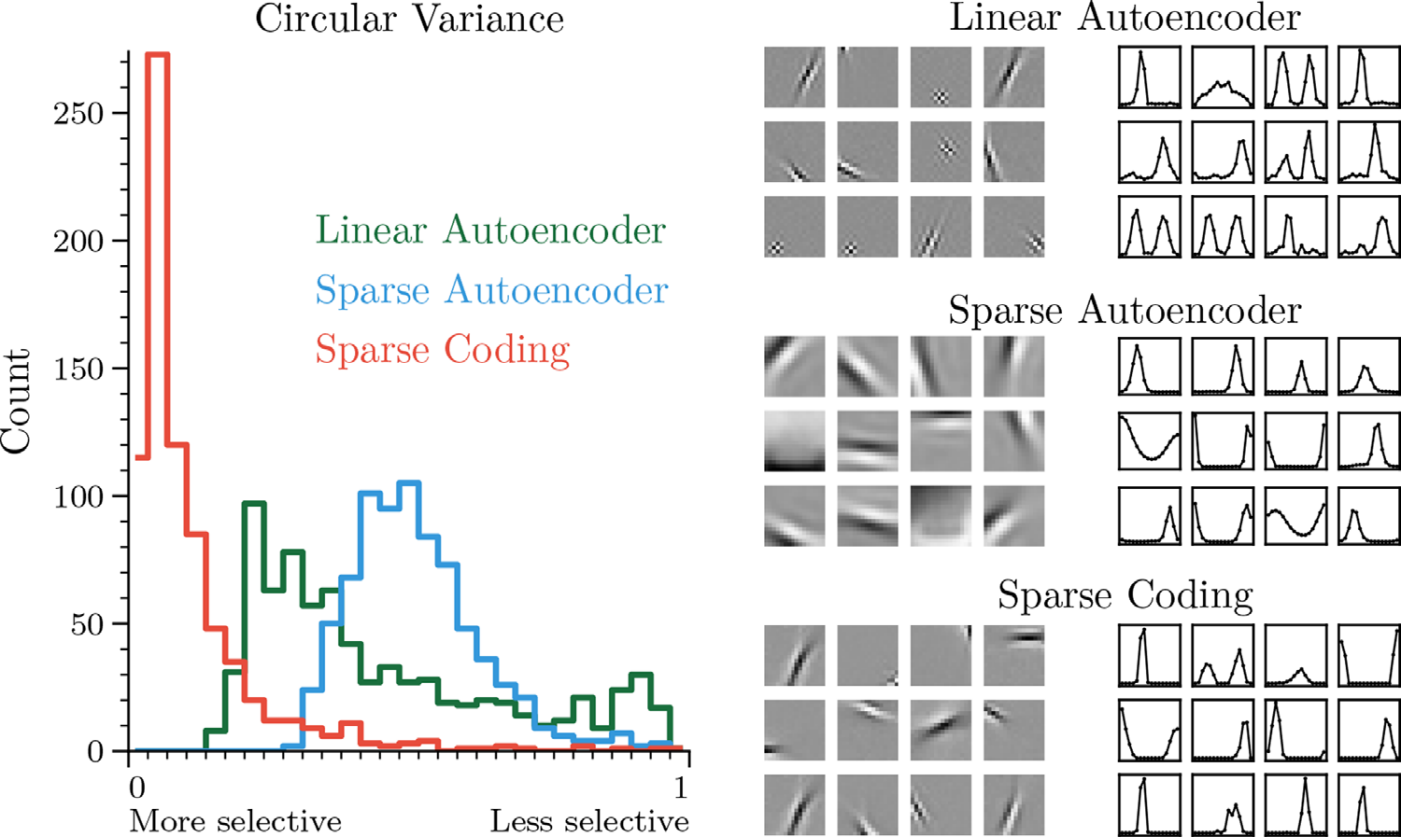
LCA neurons are more selective to oriented gratings than linear or sigmoid nonlinear neurons. On the left is a histogram of the circular variance (Ringach et al., 2002), a measure of orientation selectivity, for all of the neurons in each model. In the middle, we show a random sampling of weights learned by each model. On the right, we show corresponding orientation response curves. For the response curves, the horizontal axis represents the angle of the input stimulus, which varies from 0 to π. The vertical axis is the response of the neuron and has been normalized by dividing the response by the maximum across the 36 neurons shown. All networks received 256 pixel inputs and have 768 latent units.

Orientation selectivity is a distinguishable feature of the response properties of simple cells in Layer 4 of V1. However, since the discovery of orientation selectivity (Hubel & Wiesel, 1959), the mechanism for the computation has remained unclear. We trained three network types on one million natural image patches (van Hateren & van der Schaaf, 1998): reconstruction independent components analysis (Linear Autoencoder; Le, Karpenko, Ngiam, & Ng, 2011), a sparse autoencoder with pointwise sigmoid nonlinearities (Sparse Autoencoder; Ng, 2011), and LCA (Sparse Coding; Rozell et al., 2008) (see Appendices A.1 and A.2 for data set and network details). In accordance with typical orientation selectivity experiments, in Figure 4, we first measure the selectivity of neurons in each of these networks to full-field oriented gratings. Although all models are able to learn oriented Gabor-like filters, sparse coding exhibits a higher degree of selectivity than both the linear and pointwise nonlinear alternatives, supporting the hypothesis that lateral competition facilitates hyperselectivity. Experimental evidence recorded from V1 simple cells in monkeys (*Macaca fascicularis*) shows neurons that exhibit the full range of circular variance selectivity values for similar stimuli (Figure 1, Ringach et al., 2002), suggesting that at least some of them are more selective than what can be achieved by a single-layer pointwise linear nonlinear model.

### Natural scene selectivity

**Figure 5.**
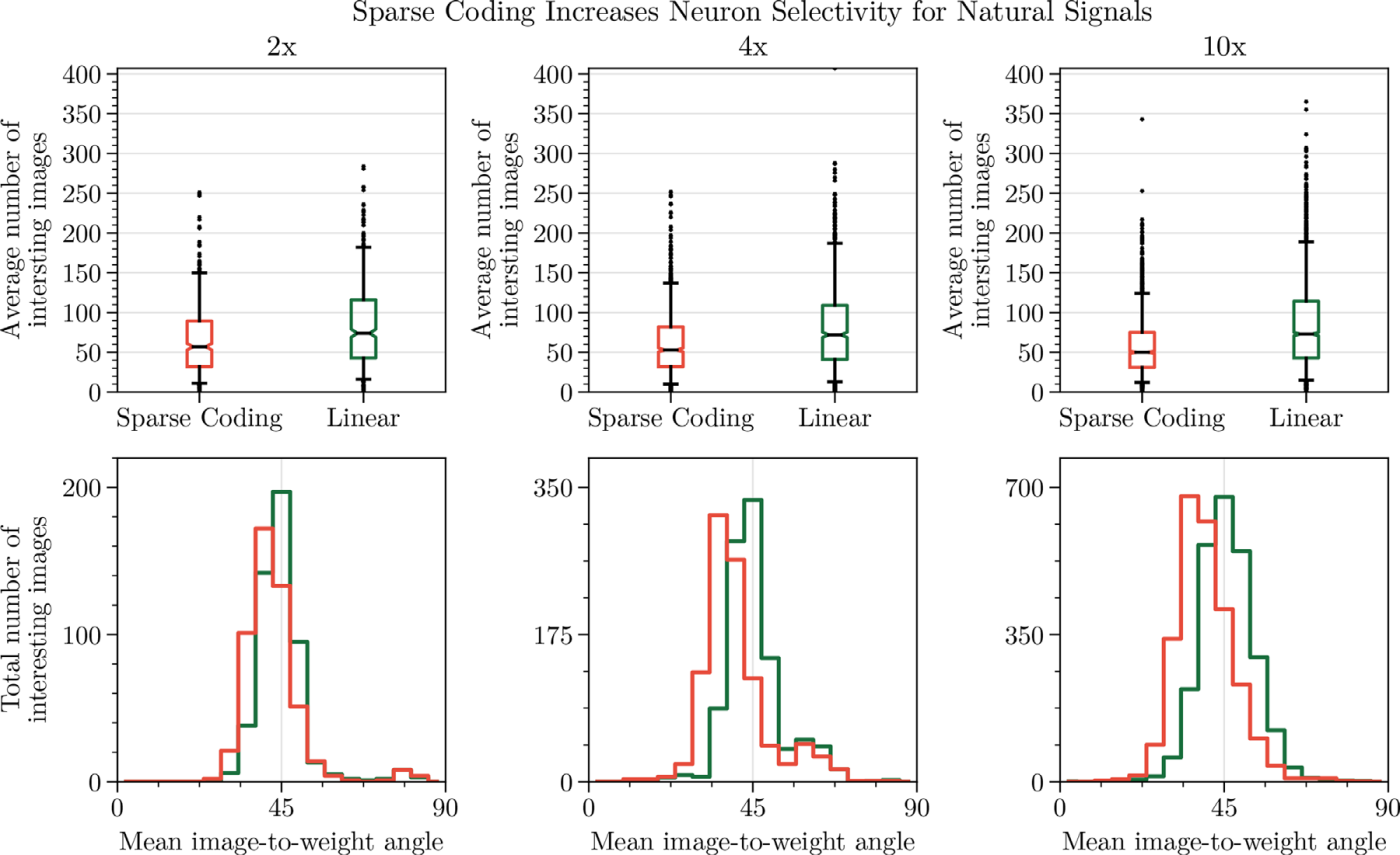
Natural image selectivity. Selected images are chosen for individual neurons by selecting those that evoke at least 50% of the maximum activation achieved from 100,000 natural image patches. (Top) LCA neurons are excited by fewer natural image patches than linear neurons with identical feedforward weights. Additionally, increasing overcompleteness reduces the average number of selected images per neuron. The box extends from the lower to upper quartile values, the notch indicates the median number, and the whiskers indicate the 5th and 95th percentiles. (Bottom) Selected images for LCA neurons have a closer angle to their feedforward weights than linear neurons. Experimental details can be found in Appendix A.4.

A common point of confusion in the field has been the assumption that a locally oriented receptive field is a sufficient condition for a neuron to exhibit the degree of orientation selectivity that is observed in physiological studies (Daugman, 1985; Ferster, Chung, & Wheat, 1996; Bell & Sejnowski, 1997; Eichhorn, Sinz, & Bethge, 2009). However, others have demonstrated that adding a population nonlinearity improves selectivity and efficiency (Geisler & Albrecht, 1995; Sompolinsky & Shapley, 1997; Sinz & Bethge, 2009). The difference between linear and nonlinear selectivity is obfuscated by the oriented grating stimuli used to estimate neuron selectivity, which are obviously more controlled than an organism's natural visual experience. For example, a carefully designed linear filter (e.g., a highly elongated Gabor) could have narrow orientation selectivity for grating stimuli without having curved iso-response contours. On the other hand, one could easily construct a high-contrast, nonoriented stimulus that activates the linear filter by the same amount as a medium-contrast oriented stimulus. Thus, the interpretation of a single neuron's response when probed with a wider range of stimuli is considerably more ambiguous than suggested by its tuning to oriented gratings. To illustrate this, in Figure 5, we probe linear and LCA neurons with a more generic class of stimuli and find that linear neurons respond to a variety of examples that are not as well matched to their preferred stimuli. To measure nonlinear selectivity, we find images out of a set of 100,000 that achieve at least 50% of the maximum activation, which we call “selected images.” We estimate this for each neuron in an LCA network as well as a linear network with *identical* feedforward weights (i.e., the same linear computation as was used in Figure 4 but with the weights changed to be exactly the same as the LCA network). We then measure the average number of selected images per neuron for different overcompleteness levels as well as the angles between each neuron's weight vector and its selected images. We find that LCA neurons exhibit higher selectivity (as measured by the average number of selected images per neuron) than linear neurons. Additionally, we find that LCA neurons prefer images that are closer in angle to their weight vectors than linear neurons. LCA neurons also become slightly more selective as one increases overcompleteness, while the linear system exhibits approximately equal selectivity. Thus, a population nonlinear encoding process selects for images that are better matched to a neuron's receptive field.

As we illustrated in Figure 2, linear or pointwise nonlinear neuron responses do not change for input perturbations that are orthogonal to their weight vector. However, if the target neuron has exo-origin bent contours, then orthogonal perturbations from the neuron's weight vector will result in attenuation of its output. Therefore, if a neuron has exo-origin iso-response contours around an oriented stimulus direction, then it will be selective against any generic perturbation away from that orientation. We would argue that this constitutes a more generic and meaningful way to quantify the neuron's orientation selectivity than simply probing it with grating stimuli.

In the previous experiments, we showed a population nonlinear network that is selective to a narrower set of natural stimuli and grating orientations than the pointwise nonlinear network. However, the same principles should apply for stimulus perturbations that are derived to maximally change the neuron's output. In other words, selectivity to a preferred stimulus can alternatively be framed as robustness against nonpreferred stimulus perturbations. In the deep learning literature, these perturbations are termed adversarial and demonstrate a deep network's inability to learn robust representations of objects in the world.

## Robustness

### Iso-response surfaces predict adversarial directions

Generically, adversarial attacks are constructed utilizing a method for producing small changes to neural network inputs that create large, potentially targeted, differences in the network outputs. Early investigations of these attacks on deep networks were done by Szegedy et al. (2013), who framed adversarial images as a counter example to the hypothesis that deep networks are able to achieve local generalization to pixel regions in the vicinity of training examples. Work from Goodfellow, Shlens, & Szegedy (2014) presented evidence that the direction of the perturbation is more important than the specific point in space, which is further supported by the discovery of universal and transferable adversarial examples (Moosavi-Dezfooli, Fawzi, Fawzi, & Frossard, 2017; Kurakin, Goodfellow, & Bengio, 2016a; Jetley, Lord, & Torr, 2018). In this section, we show that the direction of perturbation to maximally modify a neuron's output is defined by its iso-response surface. Specifically, we adopt the iso-response analysis framework to better understand adversarial attacks on neural networks with and without population nonlinearities. We show that the response geometry of LCA neurons predicts data-aligned adversarial perturbations, resulting in semantically meaningful adversarial attacks. Finally, we provide evidence suggesting that competition via lateral connections constrains an adversary, resulting in larger perturbation magnitudes.

The variety of attack strategies, networks, and targets poses difficulties for making concrete analyses. As a starting point, consider the simple case of an adversarial attack seeking to maximize the activation of a single neuron k by means of a perturbation e to an input s. This type of attack is untargeted, in the sense that there is not a specific new value that we seek from the neuron. We can write the adversary's loss $L_{k}$ as

(9) $$\begin{array}{c} L_{k}\left(e\right)=f_{k}\left(s\right)-f_{k}\left(s+e\right),\; \end{array}$$

subject to the constraint $e\in \left\{{e:∥e∥}_{\infty }<ɛ\right\}=:\Omega$. Consider an iterative adversarial attack that performs projected gradient descent on the above loss function (Kurakin, Goodfellow, & Bengio, 2016b). That is, we compute iterates $e_{i}$ by

(10) $$\begin{array}{ccc} q_{i+1} & \;= & \eta \nabla_{e}f_{k}\left(s+e_{i}\right)+e_{i}\\ e_{i+1} & \;= & \mathrm{sgn}\left(q_{i+1}\right)⊙\min(|q_{i+1}\left|,ɛ\right)\; \end{array}$$

for a step size η>0, where the second line is performing projection onto the constraint set, Ω, via the combination of element-wise sign, multiplication, and minimum operations. Within the constraint set, then, the adversary is simply following the gradient of the activation with respect to the inputs. By reversing the sign of the loss $L_{k}$, we have that an adversary seeking to minimize the activation of a neuron moves along the negative gradient.

Without additional knowledge about the gradient field, these insights do not help predict the trajectory of the attack. However, it can be shown that all of the iso-response contours will be orthogonal to the gradient, and so the attack will travel orthogonally to those contours. Locally, the neuron's activation can be written, up to terms $O(∥e{∥}^{2})$, as

(11) $$\begin{array}{cc} f_{k}\left(s+e\right) & =f_{k}\left(s\right)+\nabla f_{k}{\left(s\right)}^{⊤}e+{O(∥e∥}^{2})\; \end{array}$$

and so, to first order, the activation is constant for directions orthogonal to the gradient, is nonconstant along non-orthogonal directions, and changes maximally along the subspace spanned by the gradient.

Put another way, we can trace any curve γ(t) along the iso-response surface passing through the input s, defined by $f_{k}\left(\gamma \left(t\right)\right)=f_{k}\left(s\right)=c$, and the derivative with respect to t of f(γ(t)) will be 0. Therefore, by the chain rule,

(12) $$\begin{array}{c} 0=\nabla_{t}\left[f(\gamma (t))\right]\; \end{array}$$

(13) 0=∇f(γ(t))⊤∇tγ(t)

and thus the path along every curve restricted to the iso-response surface is orthogonal to the gradient. This includes iso-response contours.

Combining these facts, we have that, inside the constraint set, an iterative attack on a single neuron follows a trajectory orthogonal to its iso-response contours. This allows us to predict the trajectory of an adversarial attack, up until it hits the borders of the constraint set, using knowledge of the iso-response contours. In turn, this allows us to make several predictions about the behavior of certain adversarial attacks.

**Figure 6.**
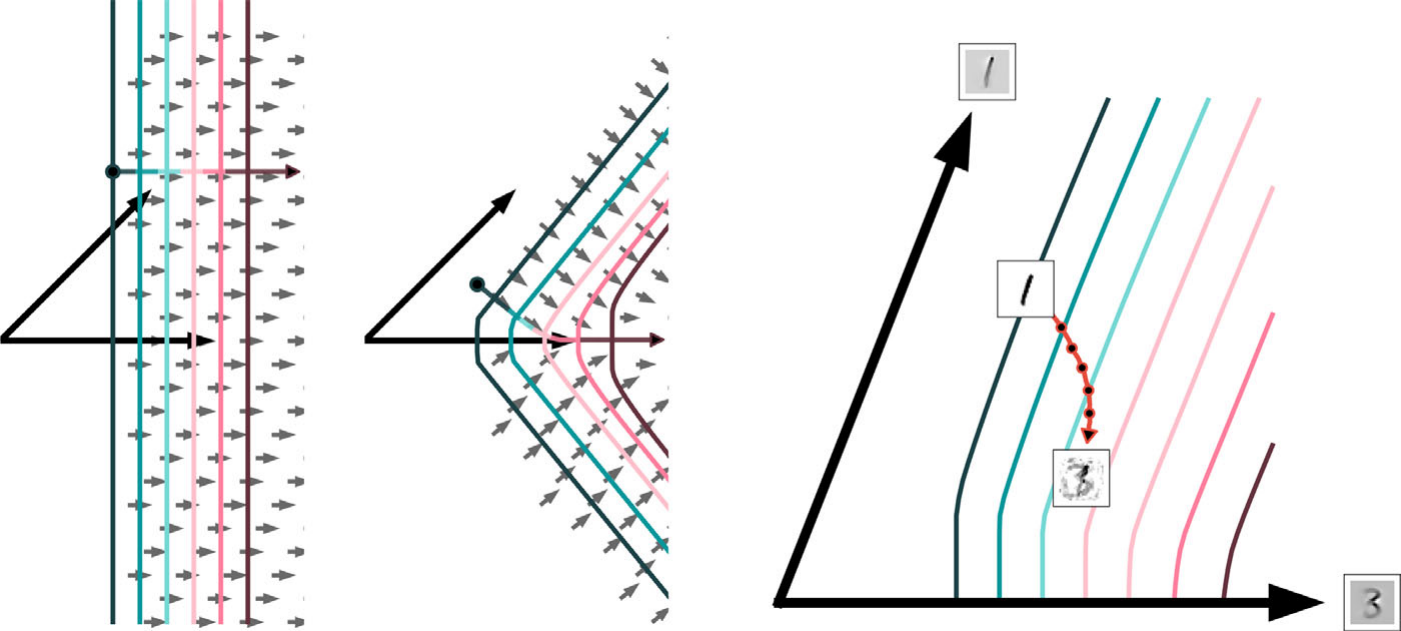
Adversarial attacks are orthogonal to iso-response contours. The left and middle plots show adversarial attacks following Equation (9) for low-dimensional models with straight and bent iso-response contours, respectively. Here, contours were computed using Euler's method. The large black arrows indicate weight vectors, the small arrows indicate gradient directions, and the colored arrow indicates the trajectory of an iterative adversarial attack against a single neuron, where color corresponds to the target neuron's activation. Note that both the attack and the gradient field are orthogonal to the iso-response contours. The right plot shows the trajectory of a projected gradient descent adversarial attack on the LCA network with 768 latent units and a linear classifier trained on the MNIST data set (the leftmost network in Figure 7). The neuron's weight vectors are displayed as images along with the input image, a 1, and the final attack output, which resembles the final classification output, a 3. The original and interim attack image positions are computed by projecting the image data onto the plane spanned by the two weight vectors.

We can predict that a gradient-based adversarial attack on a pointwise rectified neuron k will move parallel to the weight vector of that neuron, $\Phi_{k}$, since the iso-response contours are orthogonal to that vector, as depicted in the left panel of Figure 6. For a layer with population nonlinear neurons, the situation is different (Figure 6, middle panel). The adversarial attack moves orthogonally to the iso-response contours, which have exo-origin curvature centered at the weight vector. The result is that the attack moves toward, and then along, the weight vector, rather than just parallel to it.

We have shown in Figure 3 that exo-origin curvature is especially pronounced in subspaces spanned by pairs of weight vectors. The sparse coding objective used to train LCA encourages these weight vectors to collectively span subspaces that come very close to data points. This suggests that in the vicinity of samples from the data distribution, where adversarial attacks start, the effects of exo-origin curvature will be particularly strong. An adversarial attack on a generic deep network containing an LCA layer will not, in general, simply seek to maximize the activation of a single neuron in that layer. However, insofar as an attack seeks to increase a target neuron's activity, it will still need to travel in a direction with positive inner product with the gradient. The results of Figure 3 indicate that almost all of these directions will point toward the weight vector whose activity is being increased, due to the near-ubiquity of exo-origin curvature in data-relevant planes. It is not shown in the figure, but the opposite is also true—to decrease activity, an attack must be away from the the target neuron's weight vector. Although additional analysis is required, we predict that this principle will hold for gradient-free attacks (Brendel et al., 2017; Rauber, Brendel, & Bethge, 2017), which still must produce perturbations that influence individual neurons.

Combined, these findings allow us to make concrete predictions about the behavior of attacks on networks that contain an LCA layer. Due to the presence of exo-origin curvature, adversarial attacks will need to move closer to the weight vectors of neurons whose activations must be increased to obtain the same adversarial effect as attacks on a network whose layers lacked exo-origin curvature, for example, a network with only pointwise nonlinear layers. The strength of this effect will be determined by the ability of the LCA neurons to effectively span the high-density regions of the data distribution and the extent to which the actual target of the attack relies on the activation of any given neuron or set of neurons being maximized. In other words, a single LCA layer will not be as effective when paired with deeper classifiers or more complicated data sets. We believe this begs for the development of deeper generative architectures with population nonlinearities.

The third panel of Figure 6 shows several of these predictions borne out in a concrete, realistic example. It depicts an adversarial attack on a classification network composed of a linear classifier on top of an LCA layer, both trained on the MNIST data set (LeCun, 1998; data set, model, and attack details are described in the Appendix). As in Figure 2, the two weight vectors depicted as arrows define a plane. The trajectory of an adversarial attack projected onto that plane is plotted, along with the original image, classified correctly as a “1,” and the final adversarially perturbed input, classified with 90% confidence as a “3.” Note the similarity of the adversarially perturbed input to both the target class and to the weight vector (pictured at the tip of the arrows). Furthermore, the attack begins travelling in a direction approximately orthogonal to the iso-response contour in this plane indicating that, for the early phase of the attack, the single-neuron attack approximation is good. In the following section, we demonstrate that attacks against this same network also require increased perturbation magnitudes for equal adversarial confidence than attacks against a more typical pointwise nonlinear network. We find that this result holds for both the MNIST and grayscale CIFAR-10 classification data sets.

### Sparse coding provides defense against adversarial attacks

To test how population nonlinearities affect more typical adversarial attacks, we trained fully connected, leaky ReLU (Maas, Hannun, & Ng, 2013) discriminative models on the MNIST and grayscale, cropped CIFAR-10 data sets (with preprocessing detailed in Appendix A.1) as our control (denoted “w/o LCA”). Our comparison model is an LCA layer trained without supervision and a classifier trained on LCA activations (denoted “w/ LCA”). The LCA network was trained using the unsupervised learning rule defined in Equation (5), and the supervised classifier weights were trained by minimizing the cross-entropy between the one-hot ground-truth labels and the softmax output of the final fully connected layer. The models were controlled to have the same number of trainable weights and were trained to have comparable validation accuracy and weight convergence. We additionally matched confidence calibration (Guo, Pleiss, Sun, & Weinberger, 2017) for each classifier, so that the confidences associated with their predicted class labels equally reflected their correctness on the test set (see Appendix A.6 for additional details). We trained two-layer and three-layer networks for both data sets, where the “w/ LCA” version would have a one-layer and two-layer classifier, respectively. Finally, for both the two- and three-layer networks, we varied the number of first layer neurons.

For the results shown in Figures 6 (right), 7, and 8, we conducted a random targeted gradient descent attack. Following the confidence-based attack from Szegedy et al. (2013), our attack was *unbounded* and halted once the classifier confidence in the target adversarial label reached 90%. Therefore, the attack is modified from Equation (10) to account for the target label and remove the projection step:

(14) $$\begin{array}{c} s_{i+1}^{*}={\text{Clip}}_{s^{*}}\left\{s_{i}^{*}+\alpha \text{sign}\left(\nabla_{s}L\left(s,y_{\text{target}}\right)\right)\right\},\; \end{array}$$

where $s_{0}^{*}=s$ is the unperturbed image, L(·) is the cross-entropy loss function, $y_{\text{target}}$ is a random (incorrect) label, α is the step size, and the clip operation constrains the adversarial images $s_{i}^{*}\in \left[0,1\right]$. See Appendix A.5 for additional parameter and architecture details.

**Figure 7.**
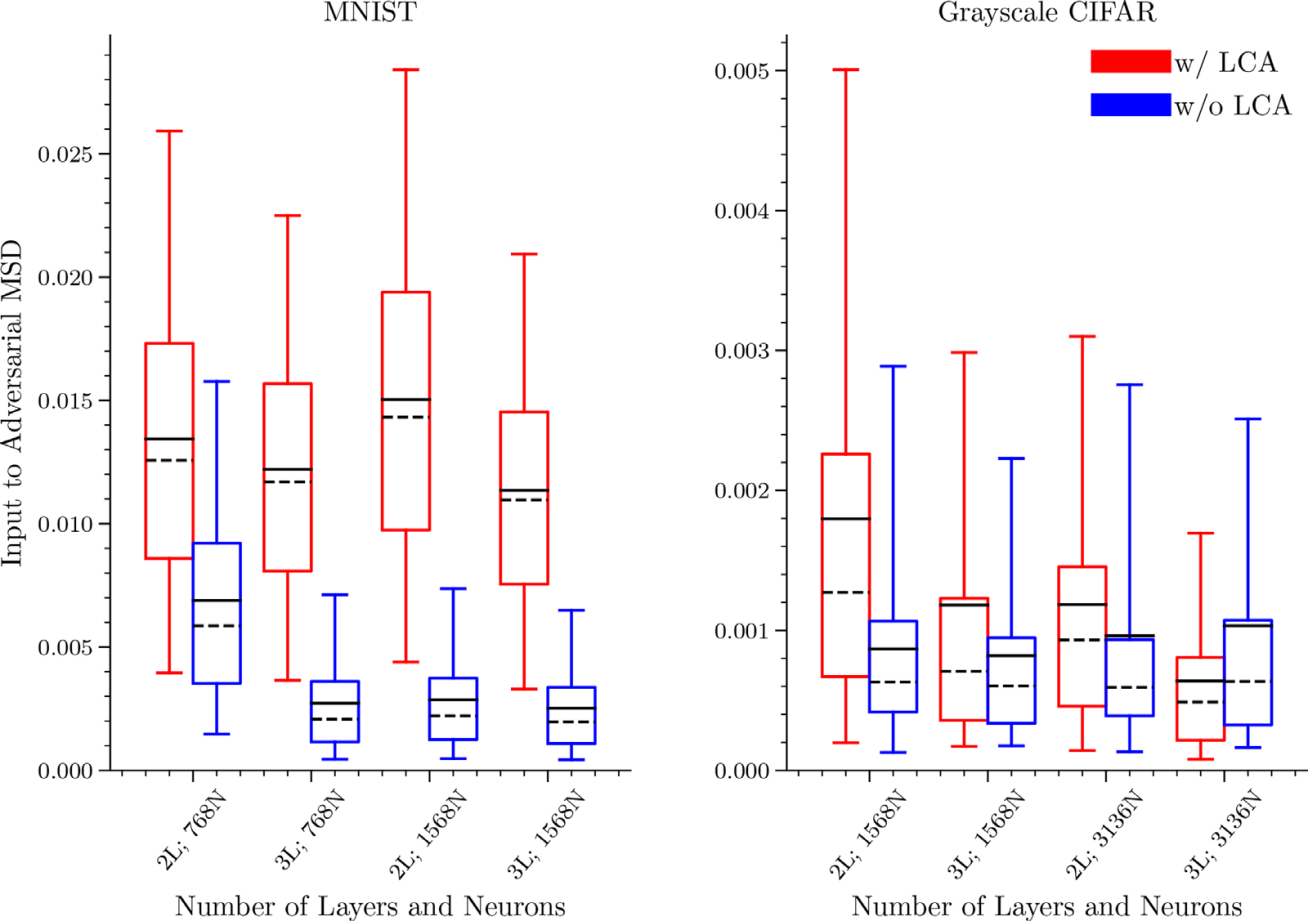
LCA protects against traditional adversarial attacks. We conducted a random targeted gradient descent attack on a two-layer network without lateral competition in the first layer (w/o LCA, blue) and with lateral competition (w/ LCA, red). The box extends from the lower to upper quartile values and the whiskers indicate the 5th and 95th percentiles for 10,000 test images from MNIST or CIFAR-10. The solid black line indicates the mean and the dashed black line indicates the median. In all but one case, the w/ LCA network outperforms the w/o LCA network in terms of the data-averaged mean squared distance between the original input and the perturbed image.

**Figure 8.**
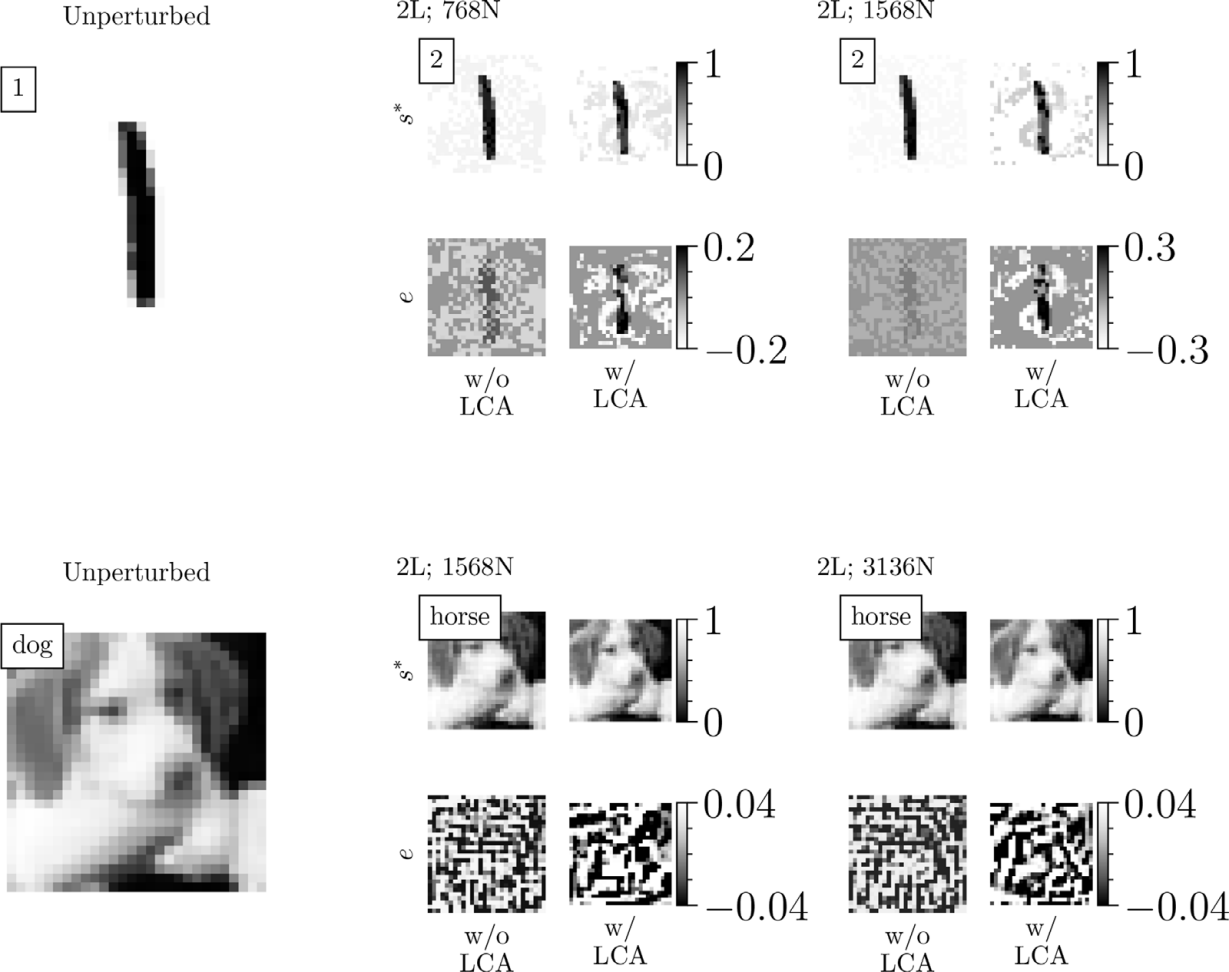
LCA influences perturbation magnitudes and directions. Images are visualizations of example data points from Figure 7. The boxed text in the top left of the images indicates the original or target label. $S^{*}$ is the input image at whatever time step corresponded to 95% classifier confidence in the target label and e is the perturbation that was added to the original image. The MNIST colors are inverted to make the perturbations more visible.

Figure 7 shows that swapping the first layer of computation with an LCA layer improves robustness against adversarial attacks. As stated previously, the amount of improved robustness achieved by incorporating LCA computation is going to be constrained by how much the classifier relies on the activation of any given LCA neuron. However, we found that the improved robustness conferred by LCA computation was still evident, albeit less pronounced, with the networks trained on grayscale CIFAR-10 as well as with deeper classifiers trained on either data set. Additionally, Figure 8 confirms that the LCA layer qualitatively influences the perturbations, and in the case of MNIST, it clearly perturbs the image toward the target category. Although we do not have a method to quantify how “semantically relevant” a perturbation is, from the digit images, one can usually identify the target class more readily in the attacks on the w/ LCA model than the w/o LCA model, as was predicted from our iso-response surface analysis. Although LCA has an impact on the perturbation for the CIFAR-10 networks, the complexity of the data makes the category relevance of the perturbation unclear. In the experiments depicted in Figure 6, we can see that a larger perturbation from the starting point of the attack is required for equal activation of the neuron. Although our analytical arguments do not directly speak to the magnitude of the perturbation, we believe the additional constraints imposed by bent iso-response contours force the attack to have increased size to achieve a given confidence criterion, which explains the results found here.

## Discussion and conclusions

Scene analysis is a challenging problem faced by biological and artificial vision systems. Fortunately, biology provides us with hints about important computational principles to solve such a problem. In this work, we investigated one such principle, recurrent inhibition, using the theoretical framework of sparse coding together with insights about the response geometry of model neurons. We first developed a scalable method for measuring model neuron iso-response and response attenuation surface curvature for high-dimensional stimuli and multiple network types. This methodology allowed us to perform a population-level response surface analysis for a large number of neurons and image planes to show that exo-origin response curvature is a general property of the LCA network. Next, we provided experimental evidence to support the hypothesis that such surface curvature connotes a higher degree of selectivity by comparing against models without surface curvature. Finally, we developed a connection between selectivity and adversarial robustness based on the geometry of the neuron response surface, and we showed that the surface can be used to predict adversarial attack directions. We also presented evidence that the exo-origin bent iso-response surface is a sufficient constraint on gradient-based adversarial attacks to result in an increase in the required perturbation magnitude to confuse a classifier with equal confidence.

Earlier work from Zetzsche and colleagues suggested that curved iso-response contours not only indicate sharper tuning but also require an overcomplete sparse coding scheme to optimally cover the signal space (Zetzsche & Krieger, 1999; Zetzsche & Röhrbein, 2001; Zetzsche & Nuding, 2005). Iso-response manifold shape has also previously been proposed to provide a direct functional interpretation of neural computation in closed-loop biological neuron recording experiments with controlled, parameterized stimuli (Gollisch & Herz, 2012). For example, Bölinger and Gollisch (2012) demonstrated that ganglion cell neurons in salamander retina that have selectivity to homogeneous receptive field contrast tend to exhibit exo-origin iso-response curvature, while neurons that lack this selectivity do not. Iso-response surface analysis was also applied to macaque V1 color tuning data by Horwitz and Hass (2012). Their models apply a pointwise nonlinearity, g, to the input pixels and then sum to produce the neuron output: $z=\sum_{i}\left(g\left(s_{i}\right)\right)$. We can think of this as equivalent to a two-layer network, which brings to light the fact that multilayer networks with pointwise nonlinearities can produce curved iso-response contours. However, we note that although a multilayer network could theoretically emulate the computation performed by the LCA (or a related) network (Hornik, Stinchcombe, & White, 1989), there is no guarantee that it will happen, as it is not measured by the typical training loss. We suggest that explicitly including population interactions as an inductive bias (Mitchell, 1980; Sinz, Pitkow, Reimer, Bethge, & Tolias, 2019) will improve computational efficiency for appropriately configured hardware and provide additional guarantees in terms of selectivity and robustness. For our experiments, we chose an LCA network as a population nonlinear function. In accordance with previous work, we believe these results to be general to networks with similar response surface curvature. However, more work must be done to use response surface geometry as a method for contrasting alternative models, such as those that explicitly implement multiplicative interactions via sigma-pi neurons (Zetzsche & Barth, 1990), competition with divisive normalization (Ren, Liao, Urtasun, Sinz, & Zemel, 2016; Sanchez-Giraldo et al., 2019), or mixed endo- and exo-origin curvature with group sparse coding (Paiton, Shepard, Chan, & Olshausen, 2020).

We provided evidence suggesting that a network with exo-origin response curvature is more selective against adversarial perturbations, a worst-case example of stimulus variations, than a network with flat response surfaces. It has also been argued that adversarial robustness is closely related to general robustness to noise perturbations, although a causal link between the two is still refuted (Fawzi, Moosavi-Dezfooli, & Frossard, 2016; Hendrycks & Dietterich, 2018; Ford, Gilmer, Carlini, & Cubuk, 2019). Here we only addressed adversarial robustness, but from our analysis, we predict that increased selectivity will result in robustness against both noise and adversarial perturbations. Indeed, a large body of work has demonstrated noise robustness for overcomplete sparse coding networks (e.g., Li & Wu, 2007; Olshausen, 2013a; Lu, Yuan, & Yan, 2013; Ahmad & Scheinkman, 2019), which complements our study to provide a more complete assessment of general robustness.

The features learned by a network are tightly linked to its adversarial examples (Goodfellow et al., 2014; Ilyas et al., 2019; Nakkiran, 2019). Much of the research on adversarial defenses has proposed modifications to the weight learning rules or to the training data to improve robustness of the network's decision boundaries (e.g., Madry, Makelov, Schmidt, Tsipras, & Vladu, 2017; Lopes, Yin, Poole, Gilmer, & Cubuk, 2019). However, the form of the computation performed by the network also influences the features learned. In addition to explicitly focusing on the features of the network, we advocate for including a recurrent synthesis step in the encoding function. This form of recurrence is suggested to facilitate Bayesian inference in the brain (Lee & Mumford, 2003; Pearl, 1988) and there exists in the literature several additional works that motivate its success as an adversarial defense. For example, humans are robust to adversarial perturbations that affect deep networks in that humans can clearly identify the correct label. However, in time-limited regimes that are suspected to result in predominantly feedforward brain computation (Thorpe, Fize, & Marlot, 1996; Serre, Oliva, & Poggio, 2007), adversarial attacks have been shown to influence human decision making (Elsayed et al., 2019), suggesting that slower recurrent computations could aid in adversarial robustness in addition to features the human visual system is selective for. Including an analysis-by-synthesis network with recurrent inference (in the form of gradient descent) as a defense method was also proposed by Schott, Rauber, Bethge, & Brendel (2018), who demonstrated provable adversarial robustness on MNIST. Our method is in the same family of models as theirs, and our theoretical arguments provide a plausible alternative explanation for their reported robustness (see Appendix A.7 for additional details). Comparing these two methods provides a critical link that suggests a key to general adversarial robustness may lie in the analysis-by-synthesis framework that is shared between them.

Recent studies on adversarial robustness have focused on the decision boundaries of classifiers, such that the attack perturbation is just large enough to push the classification decision away from the correct label (decision-based attack). We do not assess how neuron response curvature impacts the classification decision boundary location or curvature, although we recognize this connection as important for understanding adversarial vulnerability (Fawzi, Moosavi-Dezfooli, & Frossard, 2017; Moosavi-Dezfooli, Fawzi, Fawzi, Frossard, & Soatto, 2017; Moosavi-Dezfooli, Fawzi, Uesato, & Frossard, 2019). Our attack follows other works that account for model confidence (Szegedy et al., 2013; Nguyen, Yosinski, & Clune, 2015; Carlini & Wagner, 2017; Frosst, Sabour, & Hinton, 2018) (confidence-based attack). Although we do not provide decision-based attack results, other empirical work suggests that robustness in this regime can be improved with population nonlinearities, sparsity, and recurrence. For example, robustness to decision-based attacks has been shown by imposing sparsification (Marzi, Gopalakrishnan, Madhow, & Pedarsani, 2018; Alexos, Panousis, & Chatzis, 2020), recurrence (Krotov & Hopfield, 2018; Yan et al., 2019), and specifically with the LCA network (Springer, Strauss, Thresher, Kim, & Kenyon, 2018; Kim, Yarnall, Shah, & Kenyon, 2019; Kim, Rego, Watkins, & Kenyon, 2020). We offer a theoretical explanation for these findings. Congruent with these studies, we also find that our method for improving adversarial robustness does not significantly impact test accuracy and also results in more semantically relevant adversarial perturbations. We provide an accordant hypothesis that supports an alternative method for encoding information in the neural network, which will improve robustness and has no explicit bearing on the data augmentation methods or weight learning rules. Although we have not tested it, we predict that defense methods that explicitly modify the weight learning rule or data augmentation could be combined with our own method to further improve robustness.

Although there are notable exceptions, a majority of neuron models and deep neural networks still use pointwise nonlinearities due to the ease in implementation. This study makes a case for explicitly incorporating into neural computation models population nonlinearities that cause neurons to have exo-origin bent iso-response surfaces. This is not a novel perspective; for decades, researchers have identified competitive population nonlinearities as important for modeling neural function. As examples, we note that they have been utilized in neural computation models (Hopfield, 1982; Hinton & Sejnowski, 1983; Olshausen & Field, 1997; Rao & Ballard, 1999) to improve model fits to psychophysical data (Schütt & Wichmann, 2017) and V1 neuron response data (Geisler & Albrecht, 1995; Zhu & Rozell, 2013), improve generalization for image classification (Krizhevsky, Sutskever, & Hinton, 2012), and increase efficiency for image compression and storage (Ballé, Laparra, & Simoncelli, 2016; Zarcone et al., 2018). Our contribution here is to provide an analytical explanation for, and empirical evidence of, the increased selectivity to preferred stimuli and adversarial robustness enabled by inhibitory lateral connections.

## Figures and Tables

**Figure A.1.**
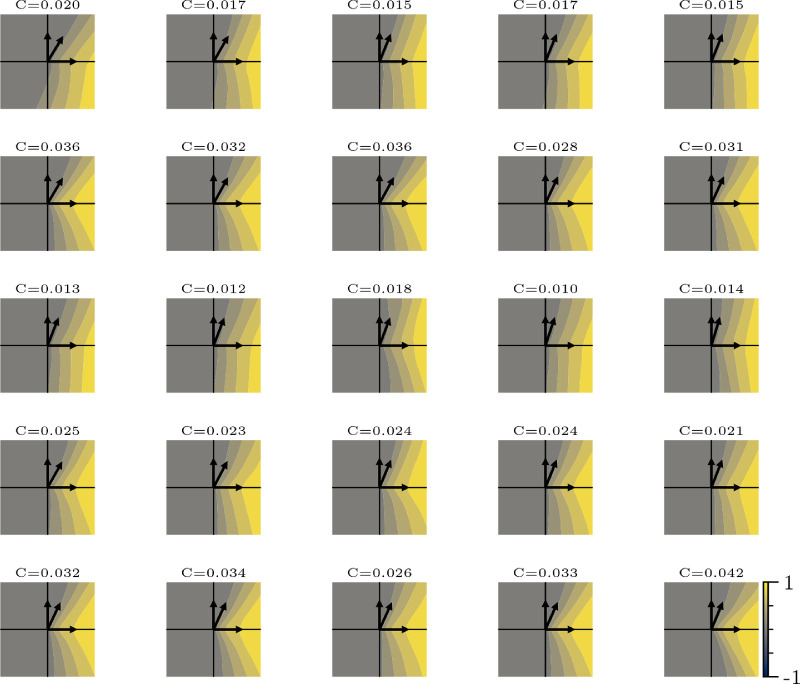
LCA response curvature. A random sampling of neurons (rows) and comparison directions (columns) for the 10× overcomplete LCA model with the corresponding curvature value.

**Figure A.2.**
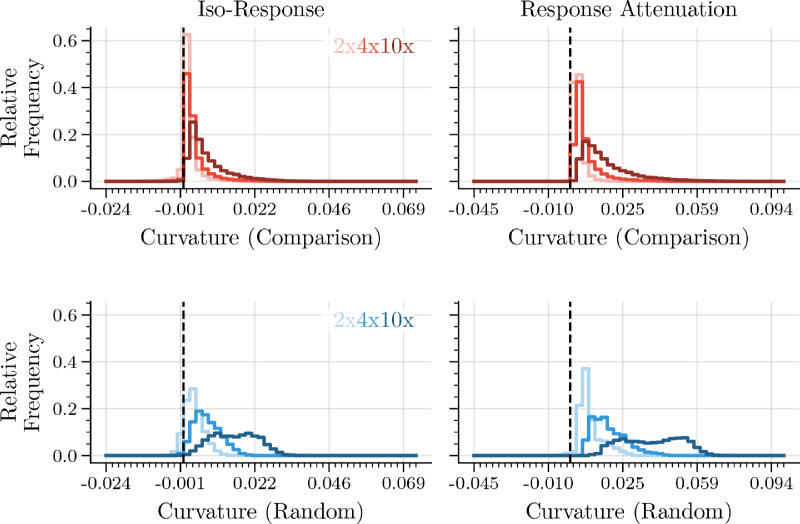
Empirically measured iso-response contours. These data are the same as in Figure 2, but with a linear scale on the y-axis.

**Figure A.3.**
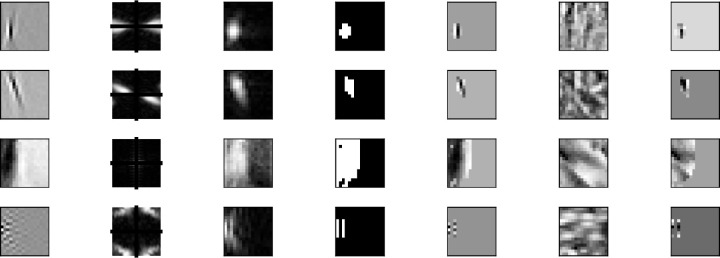
Image and weight masking procedure. From left to right, the columns indicate learned basis functions, their two-dimensional Fourier transforms, their Hilbert amplitude envelopes, thresholded envelopes converted into binarized masks, masked basis functions, natural image samples, and masked natural image samples.

**Figure A.4.**
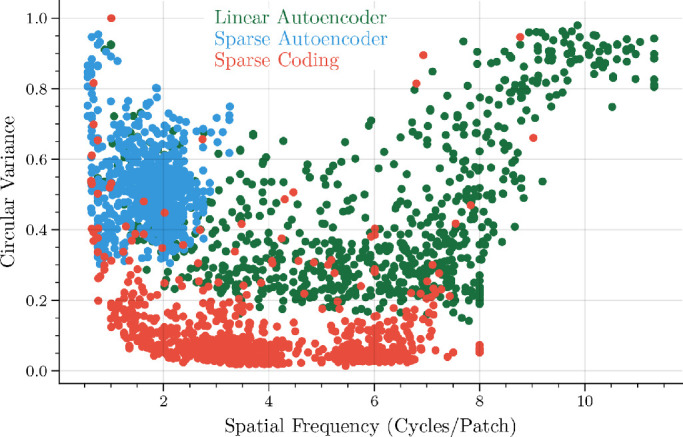
Weight spatial frequency alone does not account for improved selectivity. We compare the basis function spatial frequency against selectivity and find that the population nonlinear sparse coding network consistently shows improved selectivity (as indicated by lower circular variance) regardless of the weight spatial frequency. Each model learns unique basis functions from the same training set due to different network architectures and learning rules. Here spatial frequency is calculated by computing the radius to the peak in the two-dimensional Fourier transform of the basis function. Each dot indicates a basis function.

**Figure A.5.**
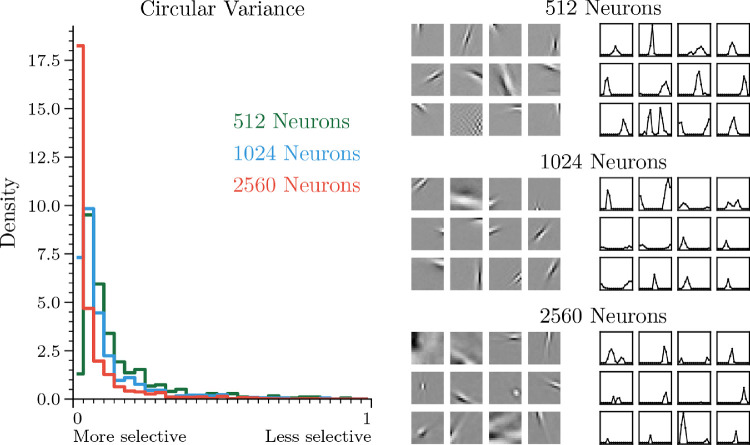
Comparing network overcompleteness against selectivity. The amount of orientation selectivity that is gained as one increases overcompleteness is much smaller than the effect observed between types of nonlinearities. The vertical axis indicates probability density, which is normalized such that the integral over the range is 1 for each model.

**Figure A.6.**
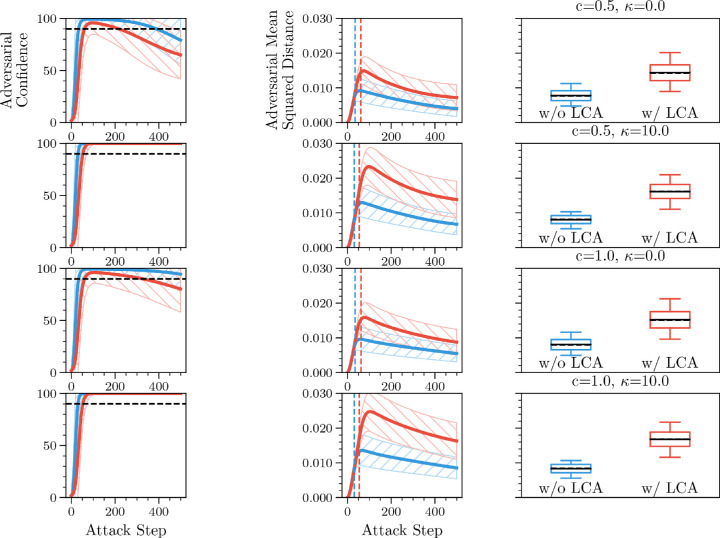
LCA network defends against an $l_{2}$ regularized attack. Carlini and Wagner (2017) describe a regularized attack (see also Equation (19)) that produces successful adversarial perturbations with a smaller $l_{2}$ distance than the standard gradient descent attack. The two columns are results when using different regularization constants, c, which trades off between adversarial confidence and perturbation size and κ, which encourages a certain degree of adversarial confidence. Each column shows: (Left) the w/ LCA model reaches the threshold adversarial confidence (black dashed line, 90%) after more steps than the w/o LCA variant. (Middle) LCA results in larger mean squared distances (MSDs) at most time steps. The colored vertical dashed lines show the time step when each model first reached the confidence threshold. (Right) A slice of the MSD at the time step when each model first reached the confidence threshold, with the same vertical axis scaling as the middle plot and plot details as in Figure 7. For the left two plots, the lines give the mean across all 10,000 test images and the hatched lighter region is the standard deviation.

**Figure A.7.**
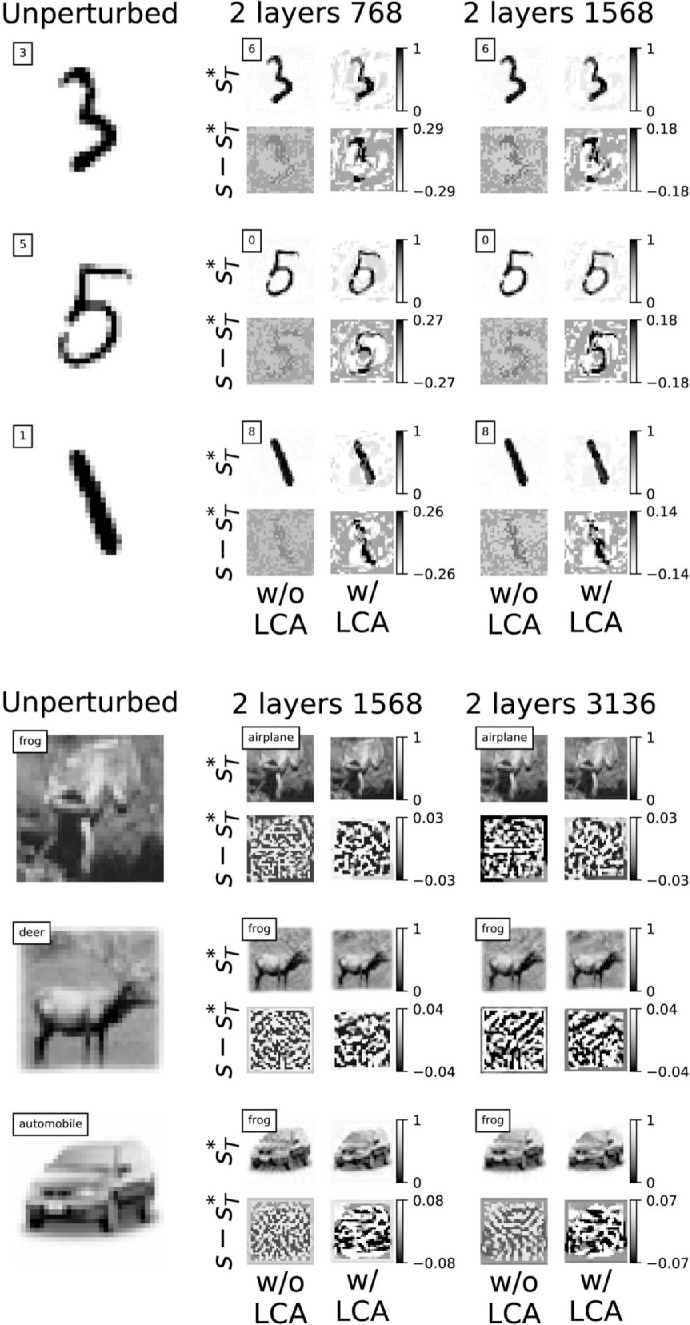
MNIST and CIFAR adversarial images. S is the original input and $S_{T}^{*}$ is the final adversarial image. In addition to larger pixel magnitudes, the w/ LCA MNIST attacks have a clear target category relevance. The CIFAR target category can not be predicted from the adversarial attacks on either model type, but there is a difference in magnitude as well as a qualitative difference in the attack structure.

## Appendix

### Appendices

#### A.1. Data preprocessing

Figures 2, 3, and 4 were all produced using data from models that were trained on the van Hateren natural scenes data set (van Hateren & van der Schaaf, 1998). The van Hateren data set was preprocessed by transforming the pixel values to log intensity, whitening, normalizing to have zero mean and unit standard deviation, and finally extracting 16-pixel ×16-pixel patches. Image whitening was done using an approximate Fourier method on the whole images (see section 5.9.3 of Hyvärinen, Hurri, & Hoyer, 2009, for an example), where we first performed a two-dimensional Fourier transform on the image, then multiplied it by a whitening filter, and finally performed an inverse Fourier transform. The whitening filter was composed by multiplying together a ramp (that has a slope of 1 and rises with frequency) component and a low-pass (starting at 0.7 times the Nyquist frequency) component.

For the MNIST data set, we individually preprocessed the images by dividing the pixel values by 255. All adversarial attacks and analysis were done after (i.e., not including) the full preprocessing pipeline.

To reduce the size of the models tested, we constructed a smaller scale CIFAR-10 data set. In order, we preprocessed the images by dividing the pixels by 255, converting each image to grayscale, subtracting the mean from each image, dividing by the per-image standard deviation, and finally cropping it to 28 by 28 pixels. For the training data, we cropped random squares, and for the test data, we cropped the centers. The adversarial attacks and analysis were back-propagated through the last three preprocessing steps, such that the mean squared distance metric used in Figure 7 was computed on 32-by-32 pixel images ranging from 0 to 1.

#### A.2. Model descriptions and training parameters

All models used in this study were fully connected. The autoencoder models used for Figures 2 and 4 had a single hidden layer with 768 units. The linear autoencoder was the RICA model from Le et al. (2011) and was trained using the following objective function:

(15) $$\begin{array}{c} \frac{1}{2}||s-\hat{s}{\left|\right|}_{2}^{2}+\lambda \sum_{i}\log\cosh u_{i},\; \end{array}$$

where $u_{i}$ is the linear output of the encoding operation: u=sW+b, s is the input image, $\hat{s}$ is the image reconstruction, b is the bias, and W is the encoding weight matrix. The ReLU autoencoder was trained using the following objective function:

(16) $$\begin{array}{ccc} L & \;= & \frac{1}{2}||s-\hat{s}{\left|\right|}_{2}^{2}+\beta_{1}\frac{1}{2}\sum_{i,j}w_{i,j}^{2}\\ & & \;+\beta_{2}\frac{1}{2}\sum_{j}{\left(1-\sum_{i}W_{i,j}\right)}^{2},\; \end{array}$$

where the last term in the objective is a normalization term that encourages the weights to have unit $l_{2}$ norm in the pixel (indexed with i) dimension, which is helpful for learning weights with localized and oriented structure. The sparse autoencoder is implemented from Ng (2011) and uses a pointwise sigmoid neuron nonlinearity. It was trained using the following objective function:

(17) $$\begin{array}{ccc} \;\;\;\;\;\;L & \;= & \frac{1}{2}||s-\hat{s}{\left|\right|}_{2}^{2}+\beta_{1}\frac{1}{2}\sum_{i,j}w_{i,j}^{2}\\ & & +\;\beta_{2}\frac{1}{2}\sum_{j}{\left(1-\sum_{i}W_{i,j}\right)}^{2}\\ & & +\;\lambda \frac{1}{2}\sum_{j}\rho \log\frac{\rho }{\hat{\rho_{j}}}+\left(1-\rho \right)\log\frac{1-\rho }{1-\hat{\rho_{j}}},\; \end{array}$$

where ρ is the target firing rate and $\hat{\rho_{j}}$ is the average firing rate of neuron j, and λ is the sparsity constraint parameter. Table 1 gives all of the parameters used for training the models on the van Hateren data set.

**Table 1.** Model training parameters for the van Hateren data set for Figures 2 and 4.

Parameter	Linear AE	ReLU AE	Sparse AE	LCA
Figure	2, top-left; 4	2, top-right	2, bottom-left; 4	2, bottom-right; 4
Number of neurons	768	768	768	768
Dropout	1.0	0.3	1.0	1.0
Learning rate	0.3	0.001	0.002	0.01
$\beta_{1}$	N/A	0.002	0.06	N/A
$\beta_{2}$	N/A	0.0001	1.0	N/A
λ	1.25	N/A	N/A	0.72
ρ	N/A	N/A	0.01	N/A

AE, Autoencoder; N/A, Not Applicable.

For Figures 3 and 5, we trained the LCA model on the van Hateren natural image data set with three levels of overcompleteness that had 512, 1,024, and 2,560 neurons. All parameters for these models were unchanged from the 768-neuron version outlined in Table 1, except for (1) λ, which was 0.55, 0.8, and 0.8 for each level of overcompleteness, respectively, and (2) the number of inference steps was increased from 75 to 120.

For the MNIST classification experiments, we used the parameters defined in Table 2. The “L-ReLU” nonlinearity is the leaky rectifier introduced by Maas et al. (2013). For all data sets, the LCA inference time constant, τ, from Equation (3), was 0.033. The CIFAR-10 supervised model parameters were largely the same, with the differences that (when it was used) the dropout keep probability for the “w/o LCA” models was increased from 0.5 to 0.8 and the supervised learning rate was 0.0005. The LCA model was always pre-trained using the parameters specified in Table 1, except that the dictionary learning rate for MNIST was 0.1 and for CIFAR-10 was 0.001.

**Table 2.** Model training parameters for the MNIST data set for Figures 7 and 8.

Parameter	w/o LCA 2-layer	w/o LCA 3-layer	w/ LCA 2-layer	w/ LCA 3-layer
Number of neurons	768; 10	768; 512; 10	768; 10	768; 512; 10
Dropout keep probability	0.5; 1.0	0.5; 0.5; 1.0	1.0; 1.0	1.0; 0.5; 1.0
Supervised learning rate	0.0001	0.0001	0.0001	0.0001
Activations	L-ReLU; Identity	L-ReLU; L-ReLU; Identity	LCA; Identity	LCA; L-ReLU; Identity

#### A.3. Curvature details and additional experiments

To compute the two-dimensional curvature planes (as in Figure 2), we use an even tiling of 30 × 30 points in a two-dimensional space with axes defined using the procedure in the second section. Each point in the two-dimensional plane is then injected into the higher-dimensional space and rescaled to have an $l_{2}$ norm that is equal to 31.7, which is the measured norm of the 1 million van Hateren training examples (after preprocessing) described above. We compute curvature using the two-dimensional points and corresponding normalized activations from these planes. We first extract points corresponding to both the iso-response and response attenuation lines. To do this, we mirror the activations in the upper-right quadrant to the lower-right quadrant. For the iso-response line, we find iso-valued contours for a target activation of 0.5 using the *measure.find_contours* function from the Python scikit-image package (Van der Walt et al., 2014). For the response attenuation line, we record activity values along a one-dimensional slice perpendicular to the $\Phi_{k}$ axis at a position that corresponds to the same target activation of 0.5. This process gives us two one-dimensional lines: The iso-response line is a set of two-dimensional coordinates corresponding to points that produce approximately the same activation, and the response attenuation line is a set of activations for a one-dimensional slice of y (ν direction) positions at a specific x ($\Phi_{k}$ direction) coordinate. We then fit both extracted lines to second-order polynomials using the *polynomial.polyfit* function from the Python Numpy package (Van Der Walt, Colbert, & Varoquaux, 2011). For each line, we use the coefficient on the squared term of the polynomial fit to quantify the curvature.

Figure A.1 gives a random sampling of neurons and comparison planes for the 10× over complete LCA network. Figure A.2 shows the same data from Figure 2 but with a linear y-axis.

#### A.4. Details and controls for the selectivity experiments

To produce the grating stimulus, we first computed the optimal spatial frequency for each neuron's weight vector by finding the peak amplitude of the vector's Fourier transform. Next, we constructed a set of full-field grating stimuli with the target spatial frequency, 16 equally spaced orientations from 0 to π radians, and 8 equally spaced phases from -π to π radians. Finally, we use circular variance to measure the orientation selectivity, which produces a bounded metric between 0, indicating increased activity (with respect to the mean) for only one of the discrete set of orientations and 1, indicating equal activation to all orientations. For each orientation, $\theta_{j}$, we computed the mean activation across phase, $a_{j}$. The circular variances are then defined as V=1-|R|, where R is

(18) $$\begin{array}{c} R=\frac{\sum_{j}a_{j}e^{i2\theta_{j}}}{\sum_{j}a_{j}}\; \end{array}$$

Many of the model neurons tested have bimodal selectivity distributions. Computing the alternative full-width at half max (FWHM) selectivity measure requires isolating a singular lobe, which is often done by hand, and fitting it with a Gaussian. Given the ad hoc nature of the FWHM measure, the lack of a complete description of response attenuation away from the preferred stimuli, and the large number of neurons tested in our experiment, we chose to use the circular variance as our metric for orientation selectivity (see Ringach et al., 2002, for a comparison between the two methods).

To measure neuron selectivity to natural images, we constructed two networks: the LCA network as described above and a linear feedforward network with identical weights (repeated for all overcompleteness levels). We compiled a test set of 10,000 random natural image patches that were not in the training set but otherwise underwent the same preprocessing as described above. For each neuron, we defined “selected images” as all images that evoked at least 50% of the maximal response from the full test set. As shown in Figure A.3, for each neuron, we constructed a binary image mask by computing the Hilbert analytic envelope of the weight (normalized between 0 and 1) and converted it to binary by setting all pixel values above a threshold of 0.5 to 1 and below to 0. We then measured the angle between the masked neuron's weight and masked interesting image vectors as a metric for how close the image was to the neuron's preferred feature.

We found that differences in learned weight spatial frequencies do not account for the improved selectivity found with the population nonlinear model. To this point, Figure A.4 demonstrates that the LCA network outperforms the pointwise nonlinear and linear networks for all spatial frequencies. We demonstrate in Figure A.5 that selectivity is increased as one increases LCA overcompleteness, although the effect is smaller than the amount gained when comparing to pointwise nonlinear models.

#### A.5. Details and controls for adversarial attacks

In Tables 3 and 4, we show the clean test accuracies for the models trained on the MNIST and CIFAR-10 data sets, respectively. In the main article, all results are for a confidence-based gradient descent attack modeled after Kurakin et al. (2016b), but where the only limiting projection is that the perturbed image is within the [0,1] pixel bounds, following Equation (14). We stop the attack per input image as soon as the adversarial confidence reaches 90%. We used standard gradient descent with a step size of α=0.005 and a maximum of 500 steps on the entire test data set with randomly chosen adversarial target labels. We found that our results were robust to a large number of different step sizes, and with the reported step size, the models always reached the confidence threshold and usually well before the 500-step limit. There have been a considerable number of adversarial defense methods published that were ultimately proven to be ineffective under careful scrutiny (Carlini et al., 2019). To ensure that the recurrent nature of the LCA model did not cause gradient obfuscation that can occur (Athalye, Carlini, & Wagner, 2018), we monitored by hand the gradient values for all models and each attack type. We additionally performed attacks with the $l_{2}$ regularized minimization method described by Carlini and Wagner (2017) on MNIST with the two-layer 768-neuron models and found minimal difference in the result, as shown in Figure A.6. The attack uses an Adam optimizer with a step size of 0.005 and a maximum of 500 steps to find a solution to the following minimization problem:

(19) $$\begin{array}{ccc} & & \text{minimize}{∥\frac{1}{2}\left(\tanh\left(s^{*}\right)+1\right)-s∥}_{2}^{2}\\ & & \;+\;c*f\left(\frac{1}{2}\left(\tanh\left(s^{*}\right)+1\right),y_{\text{target}}\right)\; \end{array}$$

where $f\left(x\right)=\text{max}\left(Z{\left(x\right)}_{i}-Z{\left(x\right)}_{t},-\kappa \right)$ for logit values Z(x) produced by the classifier at the adversarial target label index, t, and the maximum non-target label index, i. The parameter κ encourages the solver to find an adversarial instance that will be classified as the target class with high confidence for high parameter values (Carlini and Wagner, 2017). The parameter c is a trade-off between the $l_{2}$ norm constraint on the perturbation and the adversarial loss. We found comparable results when testing c∈[0.5,1.0] and κ∈[0.0,0.9,10.0]. Although we have not exhaustively addressed every method proposed for validating our results (Carlini et al., 2019), we believe our analytical support and controls provide enough confidence to warrant continued study.

**Table 3.** Test accuracy on the MNIST data set.

Model	Accuracy
w/ LCA 2-Layer 768	98.45%
w/o LCA 2-Layer 768	98.40%
w/ LCA 2-Layer 1568	98.76%
w/o LCA 2-Layer 1568	98.04%
w/ LCA 3-Layer 768	98.58%
w/o LCA 3-Layer 768	98.10%
w/ LCA 3-Layer 1568	98.60%
w/o LCA 3-Layer 1568	98.16%

**Table 4.** Test accuracy on the grayscale and cropped CIFAR-10 data set.

Model	Accuracy
w/ LCA 2-Layer 1568	56%
w/o LCA 2-Layer 1568	61%
w/ LCA 2-Layer 3136	59%
w/o LCA 2-Layer 3136	62%
w/ LCA 3-Layer 1568	68%
w/o LCA 3-Layer 1568	71%
w/ LCA 3-Layer 3136	69%
w/o LCA 3-Layer 3136	70%

#### A.6. Confidence calibration

We conducted an unbounded (i.e., limited only to be within the original pixel range of [0,1]) adversarial attack and stopped the attack once the classifiers reached a 90% softmax confidence in the adversarial label. To ensure our stopping criterion resulted in fair comparisons across classifiers, we matched confidence calibration (Guo et al., 2017) for each model using a *temperature scaling*, T, on the logits (i.e., the last network layer) before computing a softmax:

(20) $$\begin{array}{c} \text{confidence}=\text{softmax}\left(\frac{\text{logits}}{T}\right).\; \end{array}$$

Choosing an appropriate T for each classifier improves correspondence between model confidence and accuracy. This allows us to more reliably claim that the stopping confidences associated with the adversarial labels equally reflect the accuracies of the models on the test set. This is important because prediction logits can be arbitrarily scaled to result in arbitrary reported confidences without affecting accuracy. We empirically measured calibration for each model using the expected calibration error (ECE) (Naeini, Cooper, & Hauskrecht, 2015), which approximates the difference in expectation between the model's confidence and accuracy:

(21) $$\begin{array}{ccc} & & \;\;\;\;\;\;\;\;\;\;\;\;\;\;\;\text{ECE}=\sum_{m=1}^{M}\frac{|B_{m}|}{n}\left|\text{confidence}\left(B_{m}\right)-\text{accuracy}\left(B_{m}\right)\right|,\;\;\; \end{array}$$

where n indicates the number of images that fell into each of M=50 equally spaced partitioned subsets ($B_{m}$) of the test set. To match calibration for each model, we chose values for T such that corresponding ECEs were within $\frac{1}{100}$ of a unit for MNIST and $\frac{1}{10}$ of a unit for CIFAR. Table 5 reports the T values and corresponding ECEs for each tested model. When tuning the temperature, we would ideally prefer all data points to lie along the confidence-accuracy diagonal, although we were unable to achieve this for any model. Thus, we optimized for the more important criterion that the calibration between individual w/ LCA and w/o LCA pairs is as close as possible.

**Table 5.** Calibration temperature values and resulting ECE scores for all models and data sets.

Model	Data Set	Temperature	ECE
w/ LCA 2-Layer 768	MNIST	0.65	0.009%
w/o LCA 2-Layer 768	MNIST	1.0	0.007%
w/ LCA 2-Layer 1568	MNIST	0.68	0.006%
w/o LCA 2-Layer 1568	MNIST	0.5	0.005%
w/ LCA 3-Layer 768	MNIST	0.75	0.008%
w/o LCA 3-Layer 768	MNIST	1.0	0.001%
w/ LCA 3-Layer 1568	MNIST	1.0	0.008%
w/o LCA 3-Layer 1568	MNIST	1.0	0.005%
w/ LCA 2-Layer 1568	CIFAR10-Gray	0.289	2.101%
w/o LCA 2-Layer 1568	CIFAR10-Gray	0.2528	2.232%
w/ LCA 2-Layer 3136	CIFAR10-Gray	0.3	2.172%
w/o LCA 2-Layer 3136	CIFAR10-Gray	0.29	2.167%
w/ LCA 3-Layer 1568	CIFAR10-Gray	0.48	2.128%
w/o LCA 3-Layer 1568	CIFAR10-Gray	0.45	2.103%
w/ LCA 3-Layer 3136	CIFAR10-Gray	0.54	2.186%
w/o LCA 3-Layer 3136	CIFAR10-Gray	0.49	2.126%

#### A.7. A direct comparison to the analysis-by-synthesis adversarial defense

Recent work from Schott et al. (2018) proposes a verifiable adversarial defense method for the MNIST data set, which we will refer to as VAE-ABS. A crucial innovation from their work is the use of analysis by synthesis (ABS) to determine an appropriate representation from a set of class-specific generative models. The LCA network also performs ABS via a similar inference process. In summary, the key differences between the inference methods are in the latent dimensionality, sparseness of the latent code, decoder architecture, and prior. Here we will investigate the impact of the alternate decoder and prior.

The VAE-ABS architecture starts with 10 (one for each class) class-specific variational auto-encoder (VAE) networks (Kingma and Welling, 2013). When testing for adversarial robustness, for a given input image, they perform inference by minimizing the negative class-specific log-likelihood. Going forward, we will only consider the optimization process for a single class-conditioned VAE since inference is performed independently among VAEs and the arguments can be applied to each of them. We first rewrite the log-likelihood using variables that are consistent with this article:

(22) $$\begin{array}{ccc} \;\;\;\;\;\;\;\;\;\;\;L_{y}^{*}\left(s\right) & \;= & \max_{a}\log p_{\theta }\left(s|a\right)-{\text{D}}_{\text{KL}}\left[N(a,\sigma 1)||N(0,1))\right]\\ & \;= & \min_{a}\frac{1}{2}\sum_{i=1}^{N}{\left[s-f_{\text{VAE}}\left(a;\theta \right)\right]}_{i}^{2}\\ & & +\;\frac{1}{2}\sum_{j=1}^{M}\left[a_{j}^{2}+\sigma^{2}-\log\sigma^{2}-1\right]\\ & = & \min_{a}\frac{1}{2}\sum_{i=1}^{N}r_{i}^{2}+\lambda \sum_{j=1}^{M}C\left(a_{j}\right),\; \end{array}$$

where $L_{y}^{*}\left(s\right)$ is the maximum a posteriori (MAP) estimate of the log-likelihood conditioned on the class, y, and the image, s; $f_{\text{VAE}}\left(a;\theta \right)$ is the generated image for the class-specific VAE; r is the reconstruction error; ${\text{D}}_{\text{KL}}$ is the KL-divergence; σ is the conditional Gaussian standard deviation; $\lambda =\frac{M}{2}\left(\sigma^{2}-\log\sigma^{2}-1\right)$ is a constant; and $C\left(a_{j}\right)=a_{j}^{2}$. Additionally, $p_{\theta }\left(s|a\right)$ is the data likelihood, which is a function of the generative arm of the VAE network that is parameterized by θ. Comparing Equations (22) and (2) reveals that the likelihood expressions are different in the decoder function and the prior imposed on the latent variables, which manifests itself in the form of the latent variable activation cost, C(·). As is the case with our network, they compute the MAP estimate by descending the negative log-likelihood gradient:

(23) $$\begin{array}{c} \;\;\;\;\;\;\;\;\;\;-\frac{\partial L}{\partial a_{k}}=\frac{\partial f(a;\theta )}{\partial a_{k}}\sum_{i=1}^{N}{\left[s-f(a)\right]}_{i}-\lambda \frac{\partial C(a_{k})}{\partial a_{k}}.\; \end{array}$$

For our fully-connected LCA network, the decoder, $f\left(\cdot \right):=f_{\text{LCA}}\left(a;\Phi \right)=\Phi a$, is linear, and the gradient with respect to an individual latent variable, $a_{k}$, is $\Phi_{k}$. However, the VAE-ABS decoder, $f\left(\cdot \right):=f_{\text{VAE}}\left(a;\theta \right)$, is a cascade of four convolutional network layers with exponential-linear (Clevert, Unterthiner, & Hochreiter, 2015) activations, and thus the derivative, $\frac{\partial f_{\text{VAE}}}{\partial a_{k}}$, is the product of a series of piecewise linear and exponential functions. In both cases, the generated image is a function of the entire latent vector, a. This means that the each latent variable's update step is a function of the other (class-specific for VAE-ABS) latent variables. Therefore, like the LCA network, the VAE-ABS latent encoding is a population nonlinear function of the input.

Rozell et al. (2008) define the LCA network using an equivalent gradient expression that is in terms of a neuron's membrane potential and a nonlinear threshold function (our Equations (3) and (4), respectively). For the VAE-ABS defense, they perform gradient descent with the Adam optimizer directly on Equation (22). However, for the sake of comparison, we will perform the same LCA algebra steps on the VAE-ABS gradient derivation to show that the corresponding threshold function is linear. We define the LCA internal state as

(24) $$\begin{array}{c} u_{k}=a_{k}+\lambda \frac{\partial C(a_{k})}{\partial a_{k}}\; \end{array}$$

in order to arrive at the membrane update rule defined in Equation (3). The LCA membrane update can be equivalently described in terms of the reconstruction error, $\tau \dot{u_{k}}+u_{k}=\left(s-\Phi a\right)\Phi_{k}+a_{k}$ (Paiton, 2019). By adding $a_{k}$ to the right-hand side, we are able to keep the form for $u_{k}$ and $T_{\lambda }$ unchanged for the LCA network. If we rewrite this in terms of the gradient of the decoder, we get

(25) $$\begin{array}{c} \tau {\dot{u}}_{k}+u_{k}=\left[\sum_{i=1}^{N}e_{i}{\left[\frac{\partial f(a)}{\partial a_{k}}\right]}_{i}+a_{k}\right].\; \end{array}$$

As before, this equation is equivalent for the two networks as long as f(·) is taken to mean $f_{\text{LCA}}\left(a;\Phi \right)$ or $f_{\text{VAE}}\left(a;\theta \right)$. The membrane potential will also be the same as was defined in Equation (24), although for LCA $\frac{\partial C(a_{k})}{\partial a_{k}}=\text{sign}\left(a_{k}\right)$ and for VAE-ABS $\frac{\partial C(a_{k})}{\partial a_{k}}=2\lambda a_{k}$. Therefore, the VAE-ABS internal state would be $u_{k}=a_{k}+2\lambda a_{k}=3\lambda a_{k}$ and the corresponding threshold function is linear:

(26) $$\begin{array}{c} T_{\lambda }\left(u_{k}\right)=\frac{u_{k}}{3\lambda }.\; \end{array}$$

By rewriting the VAE-ABS update step in this way, we can see that a key distinction between the VAE-ABS and the LCA networks is where the inference nonlinearity comes from. In the LCA network, the nonlinearity comes from the threshold function (which in turn comes from the prior), while in the VAE-ABS network, the nonlinearity comes from the decoder.

There are some additional distinctions between their approach and ours. First, they use a Bayesian classifier with class-specific generative models, while we use a single generative model and a linear (in some experiments, nonlinear in others) perceptron classifier. They argue that adding a standard classifier on top of the VAE-ABS network would introduce adversarial vulnerability, which we agree with. Indeed, this idea is supported by Figure 7, where we can see that increasing the depth of the classifier (2L vs. 3L) decreases the advantage of using the LCA network as the first layer. Second, their class-specific generative models all use a low-dimensional (i.e., undercomplete) and dense latent code, while we use an overcomplete sparse code. They argue that their choice results in a smooth optimization space for the MAP inference process, resulting in a higher chance of reaching the global minimum and adhering to their estimates of the lower bound for adversarial examples, but they do not provide additional evidence to support this claim. We show that increasing overcompleteness increases iso-response curvature (Figure 3), and we provide a theoretical argument that suggests that curvature provides improved robustness. However, our experimental results (Figure 7) do not suggest an improvement for moderate levels of overcompleteness. We suspect that this lack of an improvement might be the result of not increasing overcompleteness enough. Note in Figure 3 that the difference in response curvature between 2× and 4× overcompleteness is quite small compared to those and the 10× overcomplete network. In the Discussion section, we also provided several other studies that suggest robustness benefits for using an overcomplete code. We identify exploring the various differences, including overcompleteness, more carefully in the context of adversarial robustness as a promising area for future research.
